# Mussel-Inspired Catechol-Functionalized Redox-Active Polypeptides for Energy Applications

**DOI:** 10.1021/acs.biomac.6c00104

**Published:** 2026-03-26

**Authors:** Shih-Guo Li, Leyla P. Gillett, Kai-Hua Mick Kuo, Soon-Mi Lim, Khirabdhi T. Mohanty, Yu-Ting Kuo, Qingsheng Wang, Alexa D. Easley, Jodie L. Lutkenhaus, Karen L. Wooley

**Affiliations:** † Department of Chemistry, 14736 Texas A&M University, College Station, Texas 77843, United States; ‡ Department of Chemical Engineering, Texas A&M University, College Station, Texas 77843, United States; § Department of Industrial & Systems Engineering, Texas A&M University, College Station, Texas 77843, United States; ∥ Department of Materials Science & Engineering, Texas A&M University, College Station, Texas 77843, United States

## Abstract

Mussel-inspired, catechol-functionalized polypeptides were synthesized from natural feedstocks and investigated as redox-active organic electrode materials, to combine electrochemical performance with sustainability and cytocompatibility. Postpolymerization grafting of dopamine onto pre-established poly­(α-l-glutamic acid)­s resulted in low conjugation, giving poor aqueous solubility and failing to function electrochemically in initial solution-state studies. Therefore, a direct strategy was adopted, whereby the redox-active catechol functionality was embedded into the monomer, followed by ring-opening polymerization to afford a polypeptide bearing a catechol group at each repeat unit. In the solid state, WAXS revealed short-range order, thermogravimetric analysis (TGA) and microscale combustion calorimetry (MCC) indicated thermal stability and low flammability, and electrochemical evaluation demonstrated a quasi-reversible catechol/*o*-quinone aqueous redox process. The composite thin film electrochemical signal intensity was significantly greater for this poly­(l-DOPA) than for the original catechol-grafted poly­(α-l-glutamic acid). Cell-viability assays further support these catechol-functionalized polypeptides as viable components in sustainable and safe energy storage media.

## Graphical abstract

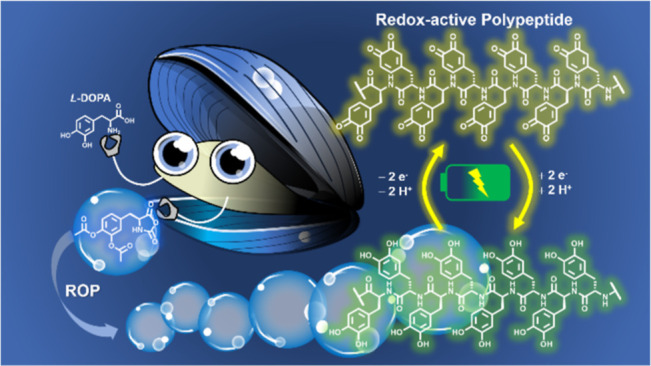

## 1 Introduction

To facilitate a more sustainable future, it is imperative to consider alternatives to conventional energy storage practices by developing materials and systems that minimize adverse effects without compromising electrochemical performance.^1,2^ Lithium ion-based platforms remain dominant in the consumer electronics market due to their high energy density, long cycle life and operational efficiency. More recently, interest has shifted toward battery technologies that offer more eco-friendly options, including sodium-ion,^3,4^ zinc-air^5^ and organic batteries.^6–9^ These platforms address concerns surrounding the ecological impact, geopolitical supply risks and safety limitations of conventional lithium ion-based materials.

Among these emerging battery chemistries, organic electrode materials may be synthesized from abundant resources and possess tunable redox-active functionalities. The inherent synthetic flexibility of organic electrode materials enables tuning of electrochemical properties while reducing dependence on rare, geographically localized and costly raw materials, making them suitable for both portable and large-scale energy storage applications.^10–13^ Additionally, organic batteries can be strategically designed from benign natural feedstocks to minimize the environmental burden while maintaining competitive energy storage capacities, making them a promising solution for sustainable energy storage technologies.^14–17^

Specifically, redox-active polymers (RAPs) have been extensively studied as active electrode materials in organic batteries, offering key advantages over small-molecule counterparts, including reduced solubility and improved cycling stability.^18,19^ Among RAPs, catechol-based polymers exhibit robust electrochemical activities, making them promising candidates as organic cathode materials. In particular, catechol-bearing polymers have high discharge potentials, fast redox kinetics and cycling stability.^20–22^ However, previous studies on catechol-containing polymers have mainly focused on their potent antioxidant,^23^ anti-inflammatory,^24^ antimicrobial,^25^ and anticancer properties;^26,27^ while their potential in energy storage remains relatively undiscovered.^28–34^ Therefore, additional exploration of the electrochemical characteristics of catechol-based polymeric materials is of interest to bridge the gap between polymer development and high-performance energy storage systems.

Few catechol-containing polymers have been explored as electrode materials in rechargeable batteries.^35,36^ Of those studied, their syntheses have relied on petrochemically derived feedstocks, which limits their sustainability and may complicate end-of-life management.^37,38^ Recently, our groups reported the syntheses of redox active polypeptides for battery applications,^6^ and advanced the anodic version of those systems to include increased components derived from biosourced and nontoxic feedstocks.^39^ Additionally, studies from other groups have reported that the α-helical backbone of polypeptides may facilitate charge and ion transport.^40,41^ Reconsidering both the side chain and backbone structures motivated the current work to expand polypeptides capable of serving as cathodic materials, in which we postulated that polypeptides constructed from catechol-containing natural products would exhibit both electroactive character and binder-like adhesion, inspired by mussel adhesive proteins.^42–45^ The dual functionality of the catechol-based polypeptide would simplify battery electrode fabrication, reduce the use of diluting binder polymer^29^ and, ultimately, pave the way for green energy solutions. Here, we present the development and characterization of synthetic polypeptides with redox-active catechol pendant groups as a step toward broadening the applications of catechol-derived materials and realizing a circular polymer economy.

## 2 Experimental Section

### 2.1 Materials

#### 2.1.1 For Synthesis

*N*,*N*-Dimethylformamide (DMF, HPLC grade), tetrahydrofuran (THF, ACS grade), ethyl acetate (EA, ACS grade), hexanes (HX, ACS grade), diethyl ether (ACS grade), petroleum ether (ACS grade), l-glutamic acid, hexylamine, dopamine hydrochloride, *N*-hydroxysuccinimide (NHS), hydrogen chloride solution 1.0 M in acetic acid, 2-aminoethanol, hydroxybenzotriazole, triethylamine and diphenylamine were purchased from Sigma-Aldrich (St. Louis, MO, USA). 4-Dimethylaminobenzaldehyde was purchased from Alfa-Aesar (Ward Hill, MA, USA). Epichlorohydrin was purchased from Fluka (Milwaukee, WI, USA). Triphosgene was purchased from Oakwood Chemical (Estill, SC, USA). Neopentylamine and 1-ethyl-3-(3′-dimethylaminopropyl)­carbodiimide hydrochloride (EDC·HCl) were purchased from TCI Chemicals (Portland, OR, USA). Acetic anhydride was purchased from Macron Fine Chemicals (Center Valley, PA, USA). Levodopa (l-DOPA) and 4-(4,6-dimethoxy-1,3,5-triazin-2-yl)-4 methylmorpholinium chloride were purchased from Chem-Impex (Wood Dale, IL, USA). HCl (1 M aqueous solution) was purchased from VWR (Radnor, PA, USA). Ethanol was purchased from Decon Laboratories (King of Prussia, PA, USA). Deuterated solvents for nuclear magnetic resonance (NMR) spectroscopy were purchased from Cambridge Isotope Laboratories (Xenia, OH, USA). DMF for polymerization and deprotection reactions was dried by passage over two columns of activated molecular sieves from a solvent purification system (J. C. Meyer Solvent Systems, Inc., Laguna Beach, CA). Neopentylamine and hexylamine were purified by stirring with CaH_2_ overnight, followed by distillation over CaH_2_ under a nitrogen atmosphere. Nanopure water was collected from a Barnstead Nanopure water system (18.2 MΩ·cm). The dialysis membrane (regenerated cellulose tubing) was purchased from Spectra/Por with a molecular weight cutoff of 3.5 kDa.

#### 2.1.2 For Electrochemical Characterization

Super P conductive carbon black was purchased from MSE Supplies (Tucson, AZ, USA). *N*-Methyl-2-pyrrolidinone (NMP) was purchased from Alfa-Aesar (Ward Hill, MA, USA). Acetate buffer (1 M, pH 5) was purchased from Spectracer (London, United Kingdom). The working, reference, and counter electrodes were purchased from Redoxme AB (Norrköping, Sweden).

### 2.2 Methods

#### 2.2.1 Synthesis of l-Glutamic Acid *N*-Carboxyanhydride (l-Glu NCA, **1**)

This procedure was followed from the literature.^46^ To a heavy wall pressure vessel, l-glutamic acid (2.0032 g, 13.6 mmol, 1.0 equiv), THF (30 mL), epichlorohydrin (ECH, 4.26 mL, 54.3 mmol, 4.0 equiv) were added sequentially under magnetic stirring. Triphosgene (2.0297 g, 6.8 mmol, ca. 0.5 equiv) was finally added in one portion, and the vessel was sealed immediately. **CAUTION: Phosgene and its derivatives are extremely hazardous.** All manipulations must be performed by experienced personnel in a well-ventilated chemical fume hood with proper personal protective equipment and necessary precautions to avoid exposure, including the use of phosgene test strips to monitor for potential exposure (test strips prepared using filter paper soaked in a mixture of 5% w/v diphenylamine and 5% w/v 4-dimethylaminobenzaldehyde in ethanol). The reaction was stirred at room temperature for 24 h (during which the suspension became homogeneous) and was filtered directly to remove any remaining unreacted l-glutamic acid. The filtrate was poured into 10-fold volume of hexanes to precipitate the crude NCA. The crude product was then collected by filtration and purified by recrystallization in petroleum ether/THF at 4 °C in a cold room to afford **1** as a white solid (1.8332 g, 78%). ^1^H NMR (400 MHz, DMSO-*d*_6_, ppm): δ 12.26 (s, 1H), 9.08 (s, 1H), 4.46 (ddd, *J* = 7.8, 5.6, 1.2 Hz, 1H), 2.36 (t, *J* = 7.5 Hz, 2H), 2.03–1.95 (m, 1H), 1.91–1.81 (m, 1H). ^13^C NMR (101 MHz, DMSO-*d*_6_, ppm): δ 173.36, 171.45, 151.91, 56.24, 29.05, 26.51. FTIR: 3370–3190, 3185–3110, 2960–2750, 2569, 2338, 2060, 1817, 1782, 1690, 1412, 1373, 1258, 1204, 1111, 1064, 975, 926, 795, 741, 663, 602 cm^–1^. MS­(*m*/*z*) [M – H]^−^ calcd, 172.0240; found, 172.0241.

#### 2.2.2 Synthesis of Poly­(α- *l* -Glutamic Acid)_40_ (P­(l-Glu)_40_, **2**)

In an argon-filled glovebox, a flame-dried 10 mL Schlenk flask equipped with a magnetic stir bar was charged with l-Glu NCA (**1**, 0.634 g, 3.66 mmol, 50 equiv), dry DMF (6 mL) and a solution of hexylamine (106 μL, 0.69 mmol/mL in dry DMF, 0.073 mmol, 1 equiv). The reaction flask was sealed with a rubber stopper, moved to a fume hood, and connected to a Schlenk line through a N_2_ flowmeter (100 mL/min) with a drying column filled with Drierite as a N_2_ outlet. The reaction was stirred for 22 h at room temperature with a stir rate of 360 rpm. The monomer consumption was monitored by ATR-FTIR spectroscopy using 2 μL aliquots sampled onto the diamond ATR plate, until the anhydride peaks (1817 cm^–1^ and 1782 cm^–1^) disappeared. Upon completion, to the resulting solution was added acetic anhydride (0.06 mL, 70 mg, 0.7 mmol, 10 equiv. relative to the amount of initiator, hexylamine) via a syringe in a dropwise manner and stirred at room temperature for another 3 h for acetyl end-capping. Finally, the mixture was dialyzed with a membrane (MWCO 3500 Da) against nanopure water for 3 d and lyophilized to obtain **2** as a white powder. In some cases, dialysis against nanopure water for 3 d was followed by dialysis against a ca. 0.1 M sodium bicarbonate solution for 2 d and then lyophilization to obtain **2**, in its sodium salt form (0.310 g, 66%). ^1^H NMR (400 MHz, D_2_O/NaOD, ppm): δ 4.24 (m), 2.19 (m), 2.03–1.78 (m), 0.78 (br). ^13^C NMR (101 MHz, D_2_O/NaOD, ppm): δ 181.50, 173.50, 53.46, 33.53, 27.89. FTIR (free acid form): 3390–3130, 3125–3000, 2990–2855, 2639, 2353, 2322, 2191, 2083, 1991, 1721, 1605, 1535, 1404, 1268, 1226, 1165, 1018, 936, 883, 787, 633 cm^–1^. FTIR (sodium salt form): 3700–3100, 3090–3000, 2990–2850, 1643, 1543, 1396, 1312, 1119, 934, 633, 602 cm^–1^. TGA in N_2_: 25–233 °C, 5% mass loss; 233–330 °C, 54% mass loss; 330–500, 18% mass loss; 23% mass remaining above 500 °C. The polymer exhibited a glass transition temperature (*T*_g_) of ca. 75 °C.

#### 2.2.3 Synthesis of Poly­(α- *l* -Glutamic Acid)_40_-*Graft*-Dopamine (P­(l-Glu)_40_-*G*-DA, 3) by Amidation

A 250 mL round-bottom flask with a magnetic stir bar was charged with P­(l-Glu)_40_ (**2**, 1.0051 g, 6.66 mmol, 1 equiv), 1-ethyl-3-(3′-dimethylaminopropyl)­carbodiimide hydrochloride (EDC·HCl, 1.5238 g, 7.95 mmol, ca. 1.2 equiv), *N*-hydroxysuccinimide (NHS, 1.8299 g, 15.9 mmol, ca. 2.4 equiv), and deionized water (125 mL). The resulting solution was stirred for 30 min in an ice bath and was allowed to warm to room temperature, and stirred for another 1.5 h. Afterward, dopamine hydrochloride (1.2173 g, 7.94 mmol, ca. 1.2 equiv) was added, and the reaction mixture was stirred overnight (20 h) at room temperature. The crude reaction mixture was preliminarily purified by dialysis with a membrane (MWCO 3500 Da) against nanopure water for 2 d and lyophilized to obtain a waxy white solid. The solid was then triturated with a small amount of DMSO and a drop of TFA, then excess water (2 ×), followed by the same procedure with DMSO/methanol and DMSO/diethyl ether (2 ×) to yield **3** as a fine, white powder (96.6 mg, 8.1%). ^1^H NMR (400 MHz, D_2_O/NaOD, ppm): δ 6.40–6.10 (m), 4.37–3.93 (m), 3.31–2.99 (m), 2.46–1.69 (m), 1.37 (br), 1.15 (br), 0.74 (br s). ^13^C NMR was attempted but provided unassignable spectrum with high signal-to-noise ratio. FTIR: 3690–3125, 3125–3010, 3000–2850, 2832, 2639, 2330, 2052, 1643, 1535, 1444, 1396, 1242, 1119, 949, 876, 795, 633 cm^–1^. TGA in N_2_: 25–215 °C, 5% mass loss; 215–347 °C, 48% mass loss; 347–500, 13% mass loss; 34% mass remaining above 500 °C. The polymer exhibited a glass transition temperature (*T*_g_) of ca. 102 °C.

#### 2.2.4 Synthesis of *O*,*O*′-Diacetyl-l-DOPA (l-DOPA­(OAc)_2_, **4**)

This procedure was followed from the literature.^47^ In a 250 mL round-bottom flask equipped with a magnetic stir bar, l-DOPA (4.9993 g, 25.353 mmol) was suspended in 100 mL of 1 M HCl in acetic acid. The suspension was stirred at room temperature under N_2_ for 2 h. Acetic anhydride (4 mL) was added to the mixture via a syringe, and the reaction was stirred for an additional 1.5 h at room temperature under N_2_. A second portion of acetic anhydride (4 mL) was added via a syringe, and the reaction mixture was heated in an oil bath at 54 °C for 1 h until the solution became clear. The mixture was cooled to room temperature and concentrated in vacuo to evaporate acetic acid and acetic anhydride, yielding a paste. Ethanol (15 mL) was added, and the mixture was stirred at room temperature under N_2_ for 0.5 h to quench remaining acetic anhydride. The crude was recrystallized from ethanol/diethyl ether at 4 °C, then washed with a copious amount of diethyl ether during filtration to afford **4** as a white solid (4.8814 g, 68%). ^1^H NMR (400 MHz, DMSO-*d*_6_, ppm): δ 8.40 (br, 3H), 7.27–7.14 (m, 3H), 4.20 (t, *J* = 6.3 Hz, 1H), 3.19–3.06 (m, 2H), 2.27 (2s, 6H). ^13^C NMR (101 MHz, DMSO-*d*_6_, ppm): δ 170.22, 168.21, 168.14, 141.81, 141.18, 133.93, 127.82, 124.50, 123.63, 52.91, 34.89, 20.42, 20.35. FTIR: 3450–2940, 2940–2335, 1736, 1597, 1489, 1427, 1366, 1196, 1111, 1018, 964, 903, 802 cm^–1^. MS­(*m*/*z*) [M + H]^+^ calcd, 282.0972; found, 282.0963.

#### 2.2.5 Synthesis of *O*,*O*′-Diacetyl-l-DOPA *N*-Carboxyanhydride (l-DOPA­(OAc)_2_ NCA, **5**)

This procedure was adapted by combining literature procedures.^46,47^ To a 150 mL pressure vessel with heavy wall, l-DOPA­(OAC)_2_ (**4**, 1.5130 g, 5.3794 mmol, 1 equiv), THF (15 mL), epichlorohydrin (1.67 mL, 21.3 mmol, 4 equiv) were added sequentially under magnetic stirring. Triphosgene (0.7937 g, 2.675 mmol, 0.5 equiv) was finally added in one portion, and the vessel was sealed immediately. **CAUTION: Phosgene and its derivatives are extremely hazardous.** All manipulations must be performed by experienced personnel in a well-ventilated chemical fume hood with proper personal protective equipment and necessary precautions to avoid exposure, including the use of phosgene test strips to monitor for potential exposure (test strips prepared using filter paper soaked in a mixture of 5% w/v diphenylamine and 5% w/v 4-dimethylaminobenzaldehyde in ethanol). The reaction was stirred at room temperature for 1.5 h, during which the suspension gradually turned clear. Upon completion, the solvent was removed by rotatory evaporation in vacuo at 45 °C and during the course, the mixture solidified as a white solid. The crude product was collected by filtration, washed with HX/EA (20 mL, 9/1, v/v), thoroughly rinsed with hexanes, and recrystallized from THF/HX to afford **5** as a white solid (1.1766 g, 71%). The NCA was dried over phosphorus pentoxide (P_2_O_5_) under vacuum and stored at −18 °C. ^1^H NMR (400 MHz, DMSO-*d*_6_, ppm): δ 9.13 (s, 1H), 7.22 (d, *J* = 8.2 Hz, 1H), 7.14–7.07 (m, 2H), 4.78 (ddd, *J* = 5.6, 5.4, 1.1 Hz, 1H), 3.09–3.00 (m, 2H), 2.26 (2s, 6H). ^13^C NMR (101 MHz, DMSO-*d*_6_, ppm): δ 170.70, 168.14, 168.08, 151.64, 141.68, 141.08, 133.81, 127.73, 124.55, 123.54, 57.96, 35.59, 20.36, 20.29. FTIR: 3540–3000, 2940, 2847, 1852, 1751, 1589, 1504, 1427, 1373, 1180, 1111, 1011, 895, 756, 679, 633 cm^–1^. MS­(*m*/*z*) [M – H]^−^ calcd, 306.0608; found, 306.0618.

#### 2.2.6 Synthesis of Poly­(*O*,*O*′-Diacetyl-l-DOPA)_50_ (P­(l-DOPA­(OAc)_2_)_50_, **6**)

In an argon-filled glovebox, a flame-dried 10 mL Schlenk flask equipped with a magnetic stir bar was charged with the NCA monomer (**5**, 0.5731 g, 1.865 mmol, 50 equiv) and dry DMF (5.7 mL). Neopentylamine (3.2 mg, 0.037 mmol, 1 equiv) in a stock solution (92 μL, 0.40 mmol/mL in dry DMF) was added directly into the monomer solution via a mechanical pipet. The flask was then sealed with a rubber stopper, moved to a fume hood and connected to a Schlenk line through a N_2_ flowmeter (100 mL/min) with a needle outlet connected to a drying tube filled with Drierite. The reaction was stirred for 17 h at room temperature with a stir rate of 360 rpm. The monomer consumption was monitored by ATR-FTIR spectroscopy using 2 μL aliquots sampled onto the diamond ATR plate, until the anhydride peak (ca. 1852 cm^–1^) disappeared. Upon completion, the mixture was purified by precipitation into diethyl ether (50 mL) with vigorous stirring. The precipitate was collected by filtration and dried under vacuum at 50 °C overnight to yield **6** as a white solid (0.3519 g, 72%). ^1^H NMR (500 MHz, DMSO-*d*_6_, ppm): δ 8.18 (br), 7.18–7.00 (m), 4.55 (m), 3.12–2.92 (m), 2.81–2.62 (m), 2.32–2.04 (m), 0.78 (br). ^13^C NMR (126 MHz, DMSO-*d*_6_, ppm): δ 170.56, 168.04, 168.02, 141.38, 140.30, 136.44, 127.20, 124.01, 122.79, 53.16, 36.81, 27.12, 20.20, 20.14. FTIR: 3450–3125, 3120–2985, 2980–2885, 1759, 1659, 1504, 1427, 1366, 1180, 1111, 1011, 957, 895, 833, 787, 648, 602 cm^–1^. Elemental analysis calcd, C:59.46, H:5.10, N:5.42; found, C:59.47, H:5.13, N:5.43. TGA in N_2_: 25–287 °C, 5% mass loss; 287–340 °C, 42% mass loss; 340–500, 11% mass loss; 42% mass remaining above 500 °C. The polymer exhibited a glass transition temperature (*T*_g_) of ca. 113 °C.

#### 2.2.7 Synthesis of Poly­(l-DOPA)_50_ (l-DOPA Polypeptide, P­(l-DOPA)_50_, **7**)

A 10 mL Schlenk flask equipped with a magnetic stir bar was charged with poly­(*O*,*O*′-diacetyl- *l* -DOPA)_50_ (**7**, 200 mg, 0.760 mmol, 1 equiv). The flask was sealed with a rubber septum, degassed by three pump–refill cycles using a standard Schlenk line with N_2_ gas, followed by the addition of dry DMF (2 mL) via a syringe. The reaction mixture was subjected to sonication under an inert atmosphere until the polymer was fully dissolved in dry DMF. 2-Aminoethanol (230 μL, 3.81 mmol, 5 equiv) was added via a syringe, and the reaction was stirred for another 7 h at room temperature under nitrogen. Upon completion, the mixture was precipitated into 1 M HCl_(aq)_ (15 mL, sparged with nitrogen for 15 min prior to use), and the polymer was collected by filtration and dried under vacuum at room temperature overnight to afford **7** as a light pink solid (100.2 mg, 72%). ^1^H NMR (500 MHz, DMSO-*d*_6_, ppm): δ 8.58 (br), 7.92 (br), 6.75–6.30 (m), 4.35 (m), 2.85–2.72 (m), 0.74 (br). ^13^C NMR (126 MHz, DMSO): δ 171.15, 144.59, 143.52, 128.71, 119.99, 116.69, 115.19, 54.07, 36.97. FTIR: 3690–2970, 1605, 1520, 1443, 1358, 1250, 1196, 1111, 957, 887, 795, 610 cm^–1^. Elemental analysis calcd, (w/23H_2_O) C:56.51, H:5.63, N:7.40; found, (w/23H_2_O) C:56.30, H:5.24, N:7.75. TGA in N_2_: 25–284 °C, 5% mass loss; 284–335 °C, 26% mass loss; 335–500, 16% mass loss; 53% mass remaining above 500 °C. DSC was attempted but did not exhibit a *T*_g_ value in the tested temperature window (50–280 °C).

### 2.3 Electrochemical Characterization and Composite Electrode Fabrication

A composite electrode of polypeptide **7** was prepared by sonicating a mixture of Super P conductive carbon black (50 wt %) and l-DOPA polypeptide **7** (50 wt %) in NMP (1 mL per 40 mg of solids) until a homogeneous ink was obtained. After homogenization, 0.5 μL of the slurry was drop-cast onto a glassy carbon electrode (2 mm diameter, active area = 0.0314 cm^2^). The electrode was left to dry under ambient conditions overnight, followed by vacuum drying at 50 °C for 24 h. The composite electrodes of polypeptide **3** and the Super P only control were prepared using the same procedure, except that the control slurry contained only carbon black (20 mg/mL in NMP). The resulting polypeptide- and Super P-coated glassy carbon electrodes (mass loading of ca. 0.64 mg/cm^2^ for polypeptide composites and ca. 0.32 mg/cm^2^ for Super P control) were utilized as the working electrodes in three-electrode beaker cells, with an Ag/AgCl (3 M KCl) reference electrode and a coiled platinum wire counter electrode. Each beaker cell contained 20 mL of degassed 1 M acetate buffer (pH 5).

### 2.4 Degradation Study

Acidic degradation study was carried out by suspending polypeptide **7** as a solid at 1 mg/mL in either 1 or 6 M HCl, followed by magnetically stirring at elevated temperature. The reaction was conducted in a vial of 40× volume vs the solution, securely clamped in an oil bath heated at 110 °C. Safety precautions included conducting the reaction inside a chemical fume hood with sash closed and using a portable blast shield. Over 24 h, the initial suspension gradually transformed into a turbid solution. The solvent was then evaporated in vacuo to afford the solidified degradation products, which were subsequently analyzed using ^1^H NMR spectroscopy, ATR-FTIR spectroscopy, and electrospray ionization mass spectrometry (ESI-MS).

### 2.5 Cell Viability Study

Mouse fibroblast cells (NIH/3T3) were purchased from ATCC (Manassas, VA) and cultured in 5% CO_2_ at 37 °C in Dulbecco’s Modified Eagle Medium (DMEM, ATCC) supplemented with 10% bovine calf serum and 1% Penicillin–Streptomycin (Sigma-Aldrich, St. Louis, MO) at Penicillin 100 U/mL and streptomycin at 100 mg/mL as the final concentrations. Cells were plated on 96-well plates at 10 k per well and cultured for 24 h in a 37 °C incubator with 5% CO_2_ prior to adding samples. P­(l-Glu)_40_-*g*-DA, **3** and P­(l-DOPA)_50_, **7** were dissolved in sterile DMSO at 10 mg/mL, filtered by a sterile syringe filter (Anotop 10, 0.1 μm), and mixed with fresh cell culture medium at dilutions over the concentration range of 100 μg/mL to 0.05 μg/mL prior to be added to cells in the 96-well plates. The plates were then incubated at 37 °C for 72 h. Subsequently, a 20-μL of CellTiter 96 AQ One Solution Cell Proliferation Assay (Promega, Madison, WI) was added to each well for the MTS assay and incubated for another 3 h. The absorbance was recorded at 490 nm by a Spectramax M5 (Molecular Devices Co., San Jose, CA) with the background measurement at 630 nm. The cell viability was calculated by comparing the readings from the control medium without cells and cells with no treatments. Data are expressed as mean ± s.d. of three determinations. ISO 10993-5 defines 70% cell viability as the threshold for cytocompatibility.^48^

## 3 Results and Discussion

Two distinct synthetic approaches were investigated for the synthesis of polypeptides bearing redox-active catechol pendant groups (Figure 1). The first was based upon our previous work that involved the construction of various polyglutamate-based backbones followed by installation of redox-active side chains.^6,39,49^ High degrees of conjugation and effective incorporation were achieved, in part, because the coupling reactions in those cases were performed distal to a well-solubilized and noncharged poly­(l-glutamate)-based backbone. In other examples of postpolymerization modification, aminolysis of benzyl-protected side chains has allowed for high degrees of functionalization.^50,51^ In this work, we were interested in converting l-glutamic acid into poly­(l-glutamic acid) followed by amidation with dopamine. Unfortunately, complications resulted in low coupling yields and poor electrochemical performance in both solution and solid state. Therefore, a second, more direct approach was developed, where the protected redox-active moiety was incorporated directly into the monomer, followed by polymerization and deprotection. This approach was more efficient, affording a polypeptide structure having a higher degree of functionalization and greater number of redox-active repeat units, affording a greater proportion of the overall mass being active and giving enhanced electrochemical signal output.

**Figure 1:**
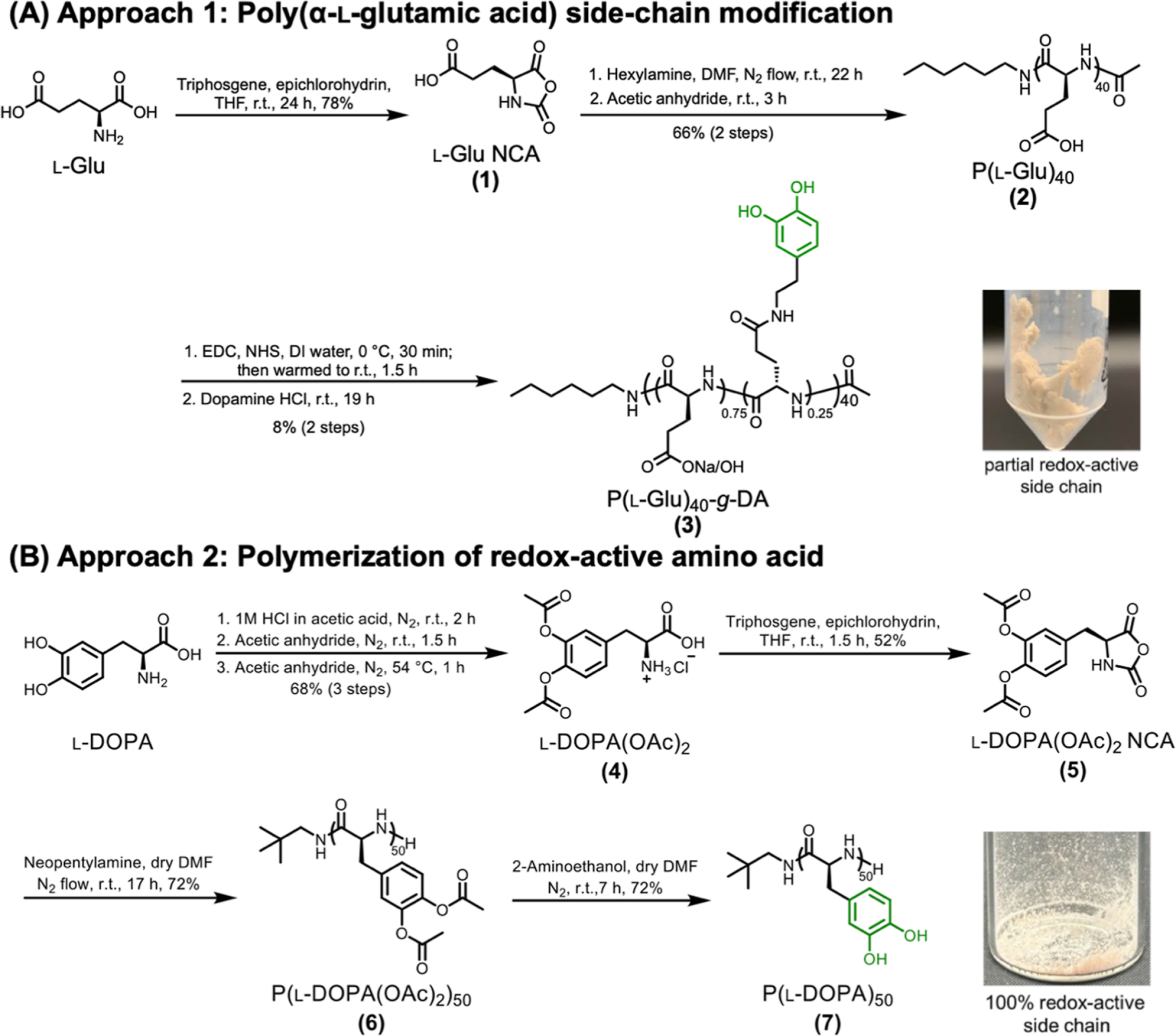
Two synthetic approaches for the preparation of catechol-functionalized polypeptides (**3** and **7**) to be used as cathode active materials: (A) Postpolymerization modification by amide coupling of dopamine to P­(l-Glu)_40_ **2** resulted in low conversion; (B) An alternative strategy utilized a protected form of l-DOPA-NCA as the monomeric building block, enabling direct incorporation of catechol moieties via NCA ROP to afford the resulting homopolypeptide **7** with high catechol functionalities.

### 3.1 Approach 1: Design and Synthesis of a Catechol-Functionalized Redox-Active Polypeptide Derived from l-Glutamic Acid and Dopamine

To enable a systematic design of sustainable redox-active polypeptide materials, l-glutamic acid has been utilized as a natural building block to construct l-glutamate–derived *N*-carboxyanhydrides (NCAs), followed by ring-opening polymerization (ROP) to construct polypeptide backbones readied for incorporation of diverse redox-active pendant groups through side-chain modifications. Previously, this approach was used to synthesize viologen-, TEMPO-, and riboflavin-functionalized polypeptides for potential use as organic electrode materials.^6,39,49,52^ Based on this concept, the synthesis of a dopamine-based cathode material was pursued from biofeedstocks, featuring an l-glutamate-based polypeptide backbone with pendant dopamine functionalities. Initial attempts focused on the ROP of the l-Glu NCA to yield a precursor polypeptide bearing a carboxylic acid group at each repeat unit, enabling subsequent installation of dopamine to introduce redox-active functionality (Figure 1A).

This first approach as outlined in Figure 1 began with synthesis of the unprotected cyclic monomer, l-Glu NCA **1** by cyclocarbonylation of l-Glu using phosgene (generated in situ from triphosgene) in the presence of epichlorohydrin as the HCl scavenger. This NCA synthesis method, based on Lu’s protocol,^46^ enabled the facile preparation of **1** without the need for side-chain protection^53,54^ or extensive purification under anhydrous conditions. The chemical structure of monomer **1** was confirmed by spectroscopic analyses, with characteristic peaks of the NH proton at 9.1 ppm and the α-CH proton at 4.5 ppm from ^1^H nuclear magnetic resonance (NMR) spectroscopy (Figure S1), together with the characteristic anhydride absorbance bands at 1817 cm^–1^ and 1782 cm^–1^ in attenuated total reflectance-Fourier transform infrared (ATR-FTIR) spectroscopy (Figure S8), collectively indicating the formation of the cyclic monomer.

The polypeptide bearing reactive carboxylic acid side chains, poly­(α- *l* -glutamic acid)_40_ (P­(l-Glu)_40_ **2**), was synthesized through N_2_ flow-assisted^55^ ROP of **1** in anhydrous DMF, with hexylamine as the initiator at a monomer-to-initiator ratio of 50:1. Reaction progress was monitored using ATR-FTIR spectroscopy, with the disappearance of the anhydride bands at 1817 cm^–1^ and 1782 cm^–1^ indicating complete monomer consumption. Acetic anhydride was then added to end-cap the ω-chain end or *N*-terminus of P­(l-Glu)_40_ **2**, and prevent undesired side reactions during subsequent amidation. Although the acetyl signals were overlapping in the ^1^H NMR spectrum and of too low intensity to be observed in the ^13^C NMR spectrum, presence of the acetyl-capped chain end was confirmed by MALDI-TOF mass spectrometry (Figure S9). A number-averaged degree of polymerization (DP_*n*_) of ca. 40 was determined from ^1^H NMR spectroscopy, based on the integration ratio of the terminal methyl protons (ca. 0.78 ppm) of the hexylamido α-chain terminus to the methine protons (ca. 4.24 ppm) of the repeat units (Figure S2).^40,41^

The catechol-grafted copolypeptide (P­(l-Glu)_40_-*g*-DA **3**) was obtained by amidation of a portion of the carboxyl groups on **2** with dopamine (Figure 1A and Figure S10).^56^ A series of reaction conditions was screened (Table S1), including variations in coupling reagents and solvent systems.^57–59^ Of the tested conditions, the best outcome was achieved using 1-ethyl-3-(3′-dimethylaminopropyl)­carbodiimide hydrochloride (EDC·HCl) and *N*-hydroxysuccinimide (NHS) in aqueous environments (entries 3 and 5 in Table S1). This method afforded **3** with an average degree of dopamine incorporation of ca. 20% across multiple repeated syntheses, as determined from ^1^H NMR spectroscopy by comparing the integration values of the aromatic protons (ca. 6.1–6.5 ppm), with those of the methine protons of the repeat units (ca. 4.2 ppm) on **3** (Figure S3). By FTIR spectroscopy, the decreased intensities of the carboxylate bands (–COO^–^) at 1543 cm^–1^ (asymmetric stretching) and 1396 cm^–1^ (symmetric stretching) in copolypeptide **3** compared to precursor **2** further suggested conversion of carboxylate functionalities to amide groups (Figures S8 and S11).^57^ Although optimization efforts modestly improved the dopamine installation, the overall conjugation efficiency remained insufficient,^60,61^ especially considering the extensive purification to remove persistent small-molecule, redox-active contaminants.

Additionally, early electrochemical measurements in an aqueous pH-buffered environment yielded a poor signal response (Figure S12), in conjunction with incomplete solubility of **3** in aqueous solution. In considering the composition of the material achieved by this first synthetic approach, the maximum percentage of polymer mass that would be comprised by the catechols would be *ca*. 40%, and at an average conjugation yield of ca. 20%, the actual catechol content was <10% of total mass. When assessing the material’s potential as an active component in organic batteries, this low redox-active component incorporation and unsatisfactory electrochemical activity would then lead to poor energy density, capacity and performance. Between this limited dopamine incorporation (structure) and solubility (properties), each of which would contribute to low concentrations of redox-active groups and the predominance of nonelectroactive repeat units, fundamental material design was reconsidered, ultimately leading to a shift in strategy toward Approach 2 of Figure 1 instead.

### 3.2 Approach 2: Design and Synthesis of a Catechol-Functionalized Redox-Active Polypeptide from l-DOPA

To overcome the hurdle associated with low conversion during postpolymerization dopamine installation, an alternative synthetic strategy was developed by employing a derivative of 3,4-dihydroxy-l-phenylalanine (l-DOPA), a naturally occurring aromatic amino acid and biosynthetic precursor of dopamine, as the monomeric building block. This alternative monomeric building block was expected to enable the direct preparation of polypeptide with superior electrochemical activity and energy storage capability (theoretical capacity of ca. 299 mAh/g) due to the inherent catechol functionality of l-DOPA that provides robust redox activity. Through a four-step process (Figure 1B), the l-DOPA-based polypeptide was successfully constructed, with a catechol group present at each repeat unit and an overall catechol content of *ca*. 65% of total mass. The synthesis involved the protection of the phenolic hydroxyl groups on l-DOPA, followed by a ring-closure reaction to form the corresponding NCA monomer.^62^ ROP of the NCA then afforded the protected P­(l-DOPA), and the final deprotection of the polypeptide side chains restored the redox-active catechol functionalities.

The NCA monomer synthesis, according to the literature procedure,^47^ proceeded in a two-step sequence, as shown in Figure 1B. Initially, l-DOPA was suspended in acetic acid containing 1 M HCl, which protonated the amino group to avoid its competitive reactivity. Subsequently, the phenolic hydroxyl groups at the aromatic 4- and 6-positions were protected with acetyl groups using acetic anhydride to afford *O*,*O*′-diacetyl-l-DOPA (l-DOPA­(OAc)_2_ **4**). Installation of the acetyl groups was confirmed by ^1^H NMR spectroscopy, with the introduction of new peaks resonating at ca. 2.27 ppm (Figure S4), and by ATR-FTIR spectroscopy, as evidenced by the appearance of the carbonyl stretching band at ca. 1736 cm^–1^ (black trace of Figure 2), consistent with the formation of ester functionalities. The cyclic monomer, *O*,*O*′-diacetyl-l-DOPA NCA (l-DOPA­(OAc)_2_ NCA **5**), was synthesized using the same method for monomer **1** construction. The NCA monomer was extensively characterized by ^1^H and ^13^C NMR (Figure S5) and FTIR (red trace of Figure 2) spectroscopies.

**Figure 2:**
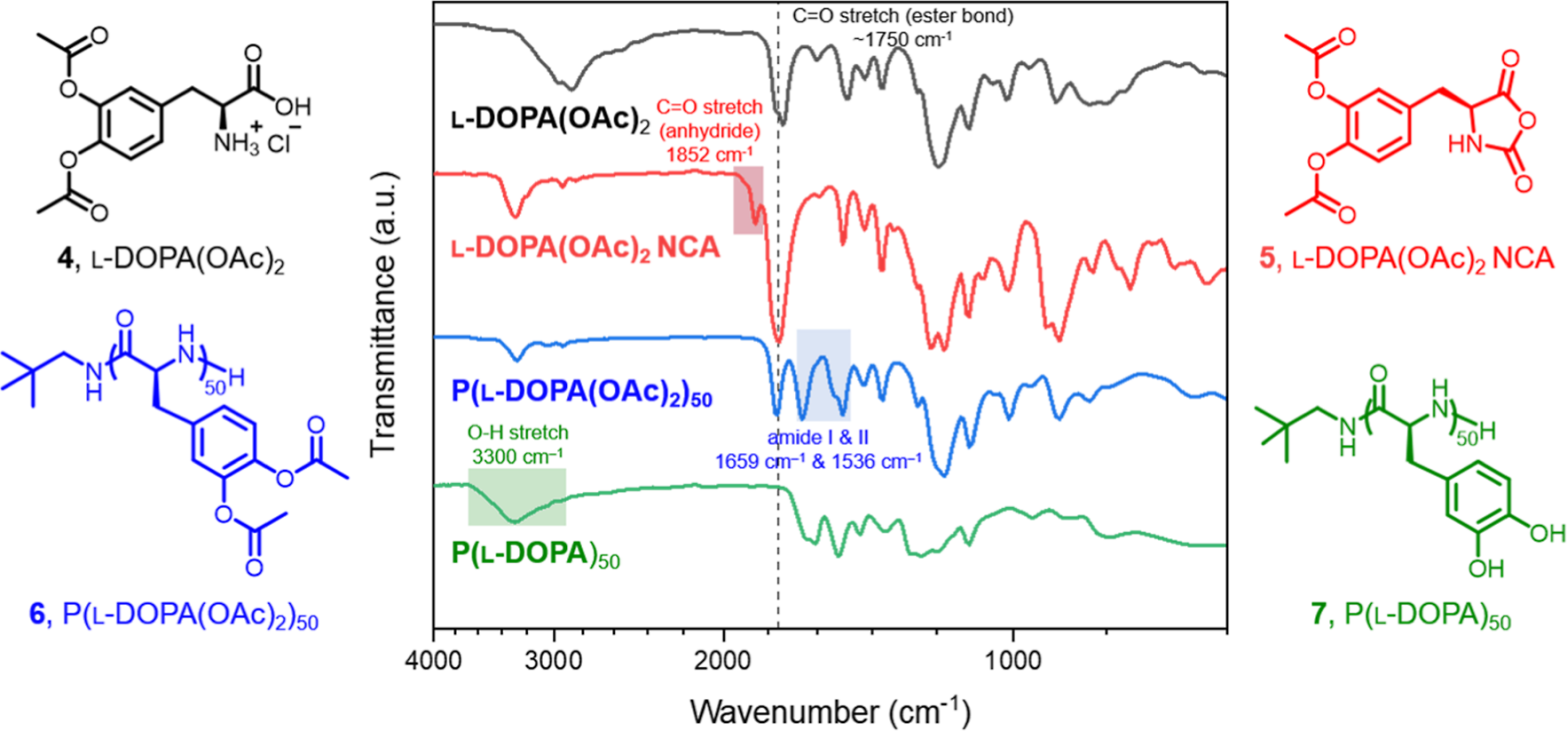
ATR-FTIR spectra of l-DOPA­(OAc)_2_ **4**, l-DOPA­(OAc)_2_ NCA **5**, P­(l-DOPA­(OAc)_2_)_50_ **6**, and P­(l-DOPA)_50_ **7**.

The polypeptide with acetyl protecting groups, poly­(*O*,*O*′-diacetyl-l-DOPA)_50_ (P­(l-DOPA­(OAc)_2_)_50_ **6**), was constructed by controlled ROP of **5** by our previously reported N_2_ flow method.^55^ Neopentylamine was used as the initiator, at a monomer-to-initiator molar ratio of 50:1. The reaction was conducted at room temperature in anhydrous DMF and was monitored by FTIR spectroscopy until the anhydride peak at ca. 1852 cm^–1^ disappeared (red trace of Figure 2) to ensure complete monomer conversion. A DP_*n*_ was estimated to be ca. 50 by ^1^H NMR chain-end analysis of **6** in DMSO-*d*_6_, by comparing the integration values of the *tert*-butyl protons (ca. 0.78 ppm, Figure 3A) on the neopentylamido α-chain terminus with the aromatic protons (ca. 7.18–7.00 ppm, Figure 3A) of the repeat units. The formation of the backbone amide linkages was further confirmed by FTIR spectroscopy, as evidenced by the amide I and II bands observed at 1659 cm^–1^ and 1536 cm^–1^ in the FTIR spectrum of **6** (blue trace of Figure 2). Based on size exclusion chromatography (SEC) analysis, polypeptide **6** exhibited a monomodal molar mass distribution with a number-average molar mass (*M*_n_) of 7.52 kDa and a broad dispersity (D̵) of 1.50 (Figure S16). The composition of polypeptide **6** was further validated by MALDI-TOF mass spectrometry, showing a dominant series of peaks with a uniform peak-to-peak separation of 263.2 Da, corresponding to the molar mass of the repeat unit (Figure S17).

**Figure 3:**
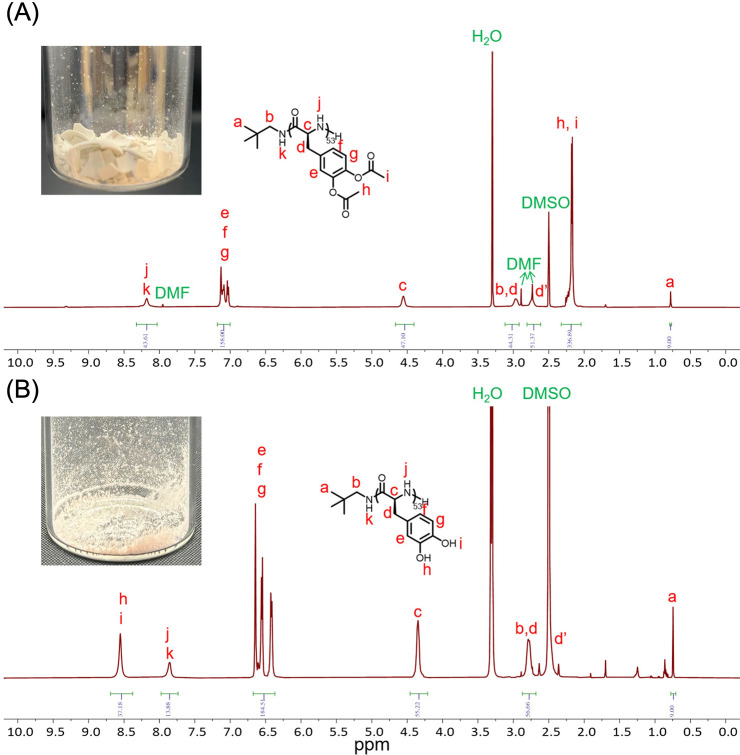
^1^H NMR spectra (500 MHz, DMSO-*d*_6_) of (A) P­(l-DOPA­(OAc)_2_)_50_ **6** and (B) P­(l-DOPA)_50_ **7**.

Deacetylation of **6** was carried out in dry DMF by aminolysis with 2-aminoethanol.^63^ To suppress catechol oxidation, the reaction proceeded under an inert (N_2_) atmosphere for 7 h, and the reaction mixture was subsequently precipitated into a deoxygenated 1 M HCl solution to afford l-DOPA polypeptide **7** as a light pink powder. The successful deprotection was confirmed by FTIR spectroscopy, with a broad hydroxyl band centered at 3300 cm^–1^ and the disappearance of the ester carbonyl stretching at 1736 cm^–1^ (green trace of Figure 2), indicating the presence of catechol groups. The chemical structure was additionally characterized by ^1^H NMR in DMSO-*d*_6_, in which the phenolic hydroxyl protons appeared as a broad singlet centered at 8.58 ppm (peaks h and i in Figure 3B). Upon deacetylation, beyond the expected upfield shifts for the aromatic proton signals, a general upfield shift in the chemical shifts was observed for more remote protons, which may be attributed to different aggregation tendencies in solution, such as increased H-bonding capability among polymer chains postaminolysis. Indeed, as noted below, the deprotected polymer exhibited lower solubility and appeared to experience greater noncovalent interactions. The UV–vis absorption behavior of **7** was investigated in DMF (Figure S18), revealing a characteristic catechol absorption centered at ca. 285 nm, corresponding to the π → π* transition of the aromatic ring.^64^ Additionally, a relatively higher intensity peak was observed near 340 nm compared to polypeptide **6**, which was attributed to a more significant *n* → π* transition.^65^

Noncovalent interactions, including π–π stacking between aromatic moieties of each repeat unit and hydrogen bonding from both the side-chain catechols and backbone amide groups, were suggested by limited solubility,^66,67^ solid-state molecular order, and properties. l-DOPA polypeptide **7** was insoluble in trifluoroacetic acid (TFA), acetonitrile (ACN), hexafluoroisopropanol (HFIP), and chloroform, also exhibiting low solubility in THF, but was soluble in more polar aprotic solvents, such as DMF, DMSO, NMP, and trimethyl phosphate (TMP). Wide-angle X-ray scattering (WAXS) patterns (Figure S19) of polypeptide **7** showed broad peaks centered at 2θ ≈ 10.1° and 20.9°, corresponding to *d*-spacings of 8.9 Å and 4.3 Å, respectively, indicative of short-range molecular ordering in the solid state. These intermolecular interactions of polypeptide **7** also translated into notable thermal stability, with thermogravimetric analysis (TGA) revealing less than 50% mass loss (53% char yield) at 500 °C, indicating strong resistance to thermal degradation (Figure S14D). Due to the high thermal stability of polypeptide **7**, the potential flame-retardant character was further investigated by microscale combustion calorimetry (MCC) testing following ASTM D7309,^68^ which showed consistently high char yields (43.3–50.0%) across three replicate tests, along with low values of total heat release (THR, 4.3–4.8 kJ/g) and peak heat release rate (pHRR, 107.4–121.4 W/g) (Table S2 and Figure S20). These results demonstrate that polypeptide **7** contains a limited fraction of combustible components, effectively suppressing rapid heat release during combustion, making it a competitive candidate among reported biobased flame retardants.^69,70^ Overall, these potential flame-retardant properties provide a foundation for battery operational safety, while also highlighting the broader potential of this polypeptide as a flame-resistant material with a molecular design strategy that could be extended to further improve safety and reliability.^71,72^

### 3.3 Electrochemical Characterization

Electrochemical studies of P­(l-Glu)_40_-*g*-DA **3** and l-DOPA polypeptide **7** were performed using a three-electrode configuration under nitrogen flow at room temperature. In all experiments, a glassy carbon electrode (GCE) served as the working electrode, a coiled platinum wire as the counter electrode, and an Ag/AgCl (3 M KCl) electrode as the reference electrode. For solution-state measurements, the polypeptide (**3** or **7**) was dissolved in 1 M acetate buffer (pH 5) at a concentration of 0.5 mg/mL, followed by sonication for ca. 5 min prior to cyclic voltammetry (CV) analysis. For solid-state studies, the working electrode was a GCE modified by drop-casting a binder-free composite slurry consisting of 50 wt % conductive carbon black (Super P) and 50 wt % polypeptide, with a mass loading of ca. 0.64 mg/cm^2^. All CV measurements were performed in an aqueous electrolyte of 1 M acetate buffer (pH 5), which was deoxygenated by sparging with nitrogen for at least 15 min before use. In control studies, the GCE coated with only super P exhibited negligible redox activity (Figure 4A), confirming that the electrochemical signals primarily originated from the catechol moieties.

**Figure 4:**
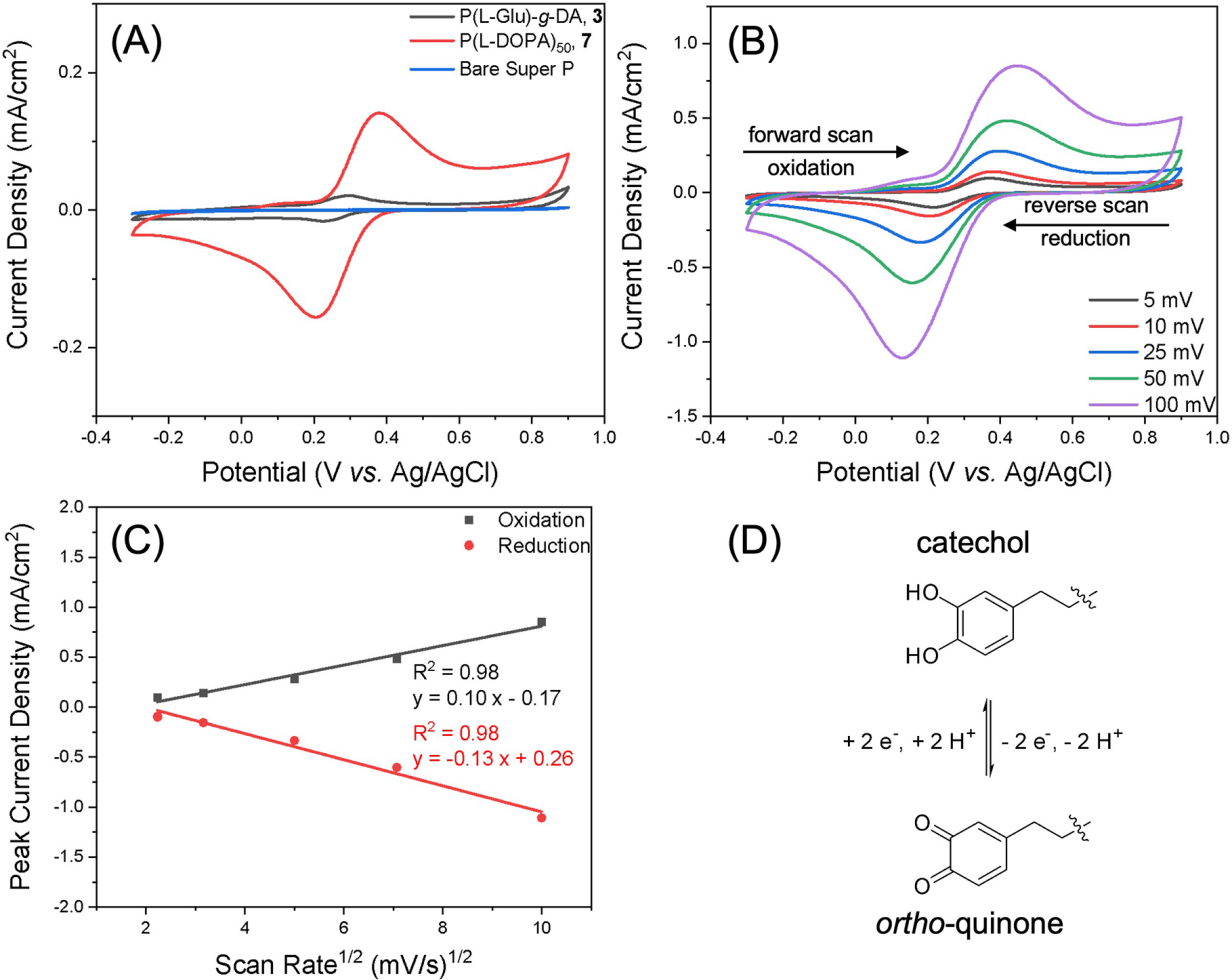
(A) Cyclic voltammograms of polypeptide composite electrodes containing P­(l-Glu)_40_-*g*-DA **3**, l-DOPA polypeptide **7**, and bare Super P carbon black at a scan rate of 10 mV·s^–1^. (B) Cyclic voltammograms of l-DOPA polypeptide **7** composite electrode collected at different scan rates (5, 10, 25, 50, and 100 mV·s^–1^), showing the second cycle at each scan rate after 10 mV·s^–1^ (3×) preconditioning. (C) Peak current density vs square-root of scan rate from the cyclic voltammograms of l-DOPA composite electrode. (D) Reversible redox reaction between catechol and *ortho*-quinone via a concerted two-proton, two-electron transfer process (PCET) in aqueous environments. CV was conducted in a three-electrode beaker cell with composite modified glassy carbon working electrode, an Ag/AgCl (3 M KCl) reference, and a coiled Pt counter electrode were used in 20 mL of degassed 1 M acetate buffer (pH 5).

As previously discussed, the synthetic design was shifted from side chain modification (Approach 1) to direct polymerization (Approach 2) to achieve better incorporation of the catechols, allowing for more robust electrochemical activity due to the increased density and relative composition of redox-active groups throughout the polymer framework. Additionally, the method of direct polymerization was expected to yield a more consistent material composition, thereby making it more suitable for scalable material and energy storage applications.

However, the early CV studies of P­(l-Glu)_40_-*g*-DA **3** and l-DOPA polypeptide **7** performed under solution state conditions showed poor signal responses (Figures S12 and S13), primarily due to the limited solubility of both polymers in the aqueous buffer solution. In contrast, under solid-state electrochemical conditions, **7** exhibited a 7-fold higher peak current density in CV compared with **3** at identical polymer mass loading (Figure 4A) and electrode composition, while maintaining similar half-wave potentials. This enhancement supports our design rationale, where the higher density of catechol units incorporated into **7** leads directly to stronger electrochemical response. Accordingly, subsequent in-depth electrochemical studies focused on the solid-state composite electrode of polypeptide **7**.

At a scan rate of 10 mV·s^–1^ (Figure 4B), the composite electrode of **7** exhibited a quasi-reversible redox couple with a half-wave potential (*E*_1/2_) of ca. 0.29 V vs Ag/AgCl and a peak separation (Δ*E*_p_) of ca. 0.18 V. The peak currents scaled with the square root of scan rate for both oxidation and reduction processes (Figure 4C), revealing the electrochemical reaction was diffusion-limited.^73^ The pair of peaks in the CV was consistent with literature reports and was attributed to the reversible reaction between catechol and *ortho*-quinone, proceeding via a concerted two-proton-coupled electron transfer (PCET) mechanism in the aqueous environment (Figure 4D).^74,75^

### 3.4 Cell Viability Study

Biocompatibility of new polymer materials is important for both the environment and human health, especially regarding long-term effects. Considering the possible applications of such batteries for wearable and implanted medical devices, such as pacemakers, the biocompatibility of battery material become of increasing significance. Cell viability testing with mouse fibroblast cells (NIH/3T3) was performed to assess the cytotoxicity of the P­(l-Glu)_40_-*g*-DA **3**, obtained by postpolymerization modification of **2**, and l-DOPA polypeptide **7**, synthesized directly from ROP of the l-DOPA-derived NCA monomer. Neither polypeptide exhibited cytotoxic effects toward fibroblast cells after 72 h of exposure. Even at a concentration as high as 100 μg/mL (achieved by transitioning from DMSO to cell media), cell viability was nearly 80%, exceeding the ISO 10993-5 cytocompatibility threshold of 70% (Figure S21).^48^ A complication to these studies and their interpretation, however, is the limited aqueous solubility of the polypeptides, which leads to limited and unknown actual polypeptide exposure to the cells. Regardless, the cytotoxicity studies overall supported the catechol-functionalized polypeptides being cytocompatible, showing no evidence of cytotoxicity. This suggests their potential use in biomedical and eco-friendly energy storage material applications, such as wearable and implantable electronic medical devices, where biocompatibility is highly valued.

### 3.5 Degradation Study

The l-DOPA polypeptide was designed with hydrolytically labile amide linkages along the backbone. With this consideration, an acidic degradation study was carried out by suspending polypeptide **7** as a solid at 1 mg/mL in either 1 or 6 M HCl, followed by magnetically stirring at elevated temperature. The reaction was conducted in a vial of 40× volume vs the solution, securely clamped in an oil bath heated at 110 °C. Safety precautions included conducting the reaction inside a chemical fume hood with the sash closed and using a portable blast shield. Over 24 h, the initial suspension gradually transformed into a turbid solution. The solution was then evaporated in vacuo to afford the solidified degradation products.

The composition of the degradation mixture was analyzed using a combination of spectroscopic and spectrometric techniques (Figure 5A). The retention of an amide absorption band in the FTIR spectrum indicated incomplete hydrolysis of the polypeptide backbone (Figure S22). To investigate whether the l-DOPA building block was regenerated during the acidic treatment of **7**, the mixture was redissolved in D_2_O, filtered through a 0.22 μm hydrophilic PVDF syringe filter, and subjected to ESI-MS analyses (Figure 5B), which confirmed the presence of l-DOPA within the degradation products. In addition to the inherent relative stability of amides, the limited extent of hydrolytic degradation was attributed to hydrophobic π–π stacking and hydrogen bonds within the material, both of which synergistically promote conformational rigidity and effectively hinder water permeation, thereby suppressing hydrolysis.^76–78^

**Figure 5:**
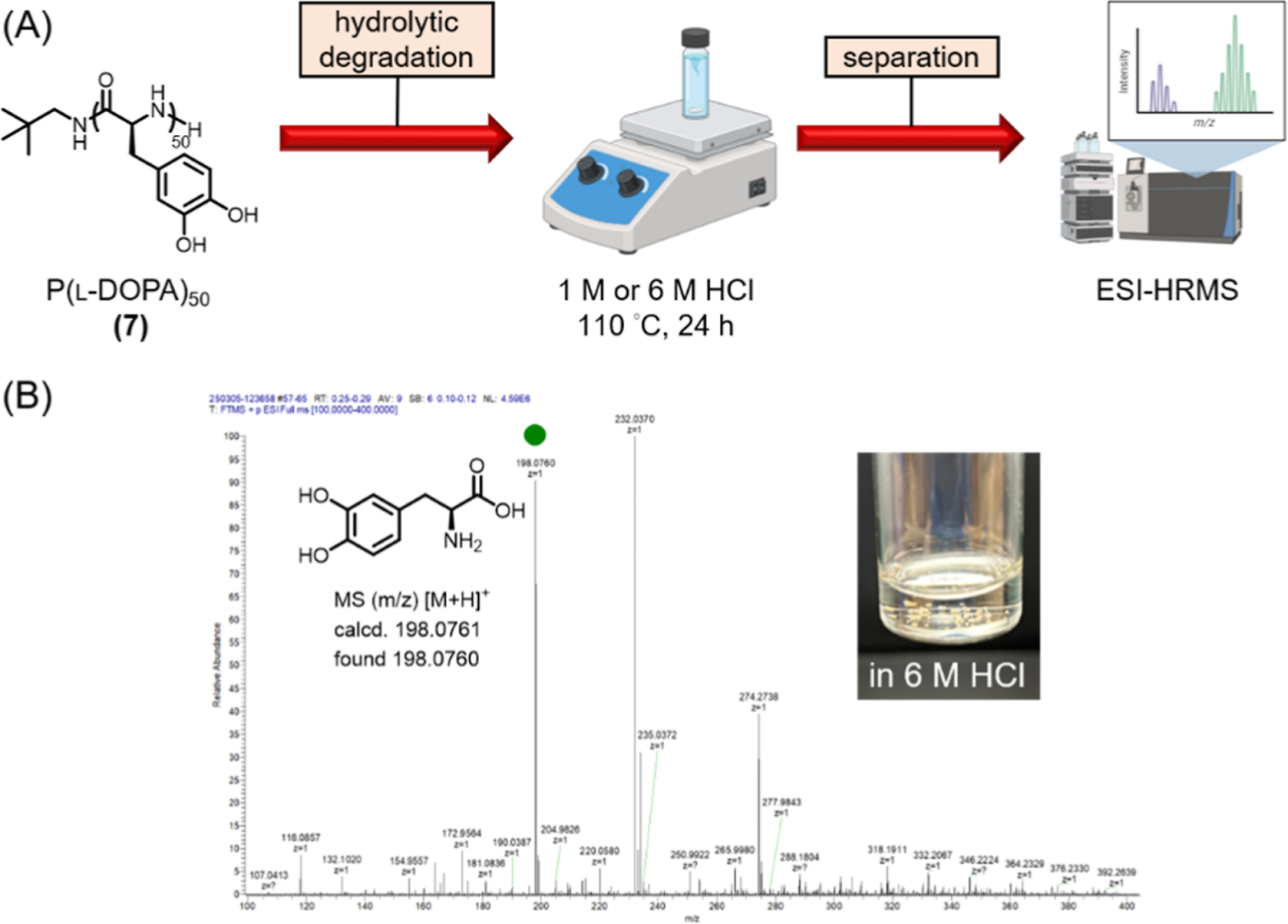
Hydrolytic degradation and analysis of l-DOPA polypeptide **7** under acidic conditions. (A) Schematic workflow depicting the acidic hydrolysis of **7** at elevated temperature, followed by isolation and compositional analysis. The figure was created with BioRender.com. (B) Mass spectrum of the D_2_O-soluble degradation products of **7** (positive ESI). The inset photo shows the residual solid after treatment in 6 M HCl at 110 °C for 24 h, indicating incomplete hydrolysis.

## 4 Conclusions

This work expands the scope of catechol-functionalized polymers for renewable energy storage by introducing a bioderived, redox-active material consisting of a naturally sourced polypeptide backbone with catechol pendant groups. Initially, a postpolymerization modification strategy was pursued by grafting dopamine onto poly­(α-l-glutamic acid) via amide coupling. However, the conjugation efficiency was limited to ca. 20%, which hampered the electrochemical performance. Ultimately, this synthetic strategy was reconsidered by developing an alternative approach. This new method leveraged a derivative of l-DOPA (levodopa) as the monomeric building block, enabling the direct incorporation of catechol motifs as l-DOPA units at each repeat unit by ROP of the corresponding NCA monomer. The resulting l-DOPA polypeptide exhibited quasi-reversible redox activity as a solid-state composite electrode under a PCET mechanism in aqueous media.

Beyond electrochemical function, thermal analysis revealed a high char residue and suppressed flammability, highlighting potential flame-retardant properties that could contribute to enhanced safety when applied as a battery material component, especially when coupled with the biocompatibility of the material for potential biological, implantable and/or wearable applications. Cytotoxicity assessments confirmed the cytocompatibility of the polypeptide, demonstrating its potential for sustainable energy storage applications with minimal negative environmental and biological impacts. Although acidic degradation studies revealed limited hydrolytic breakdown, this work establishes a promising foundation and molecular design strategy for the development of fully bioderived redox-active polypeptides as next-generation safe and sustainable organic cathode materials.

## Supplementary Material



## Notes

Detailed instrumentation methods, additional spectral data (^1^H and ^13^C NMR, ATR-FTIR, MALDI-TOF, TGA, DSC, UV–vis), cell viability data; and supplementary SEC, WAXS, and microscale combustion calorimetry (MCC) data for the characterization of catechol-containing polypeptides **3** and **7** (PDF)

## ⊥

Department of Chemical and Biomolecular Engineering, North Carolina State University, Raleigh, North Carolina 27695, United States
