# Supplementary material for: Mussel-Inspired Catechol-Functionalized Redox-Active Polypeptides for Energy Applications
Source: Biomacromolecules. 2026 Mar 26;27(4):2888–99. doi: 10.1021/acs.biomac.6c00104 (PMC13086030; doi:10.1021/acs.biomac.6c00104)
Supplement: Supplementary file 1 [file bm6c00104_si_001.pdf]

Supporting Information for

Mussel-inspired Catechol-functionalized Redox-active Polypeptides for Energy Applications

Shih-Guo Li,[†,⊥] Leyla P. Gillett,[†,⊥] Kai-Hua Mick Kuo,[†] Soon-Mi Lim,[†] Khirabdhi T. Mohanty,[‡] Yu-Ting Kuo,[‡] Qingsheng Wang,[‡,∝,¥] Alexa D. Easley,[‡,§] Jodie L. Lutkenhaus[‡,¥] and Karen L. Wooley[†,¥,*]

[†]Department of Chemistry, Texas A&M University, College Station, Texas 77843, United States

[‡]Department of Chemical Engineering, Texas A&M University,

College Station, Texas 77843, United States

[∝]Department of Industrial & Systems Engineering, Texas A&M University,

College Station, Texas 77843, United States

[¥]Department of Materials Science & Engineering, Texas A&M University,

College Station, Texas 77843, United States

[§]Present Address: Department of Chemical and Biomolecular Engineering,

North Carolina State University, Raleigh, North Carolina 27695, United States

[⊥]S.-G.L. and L.P.G. contributed equally to this work.

[*]Corresponding author e-mail: wooley@chem.tamu.edu

Instrumentation

Spectroscopic Characterization:

^{1}H and ^{13}C NMR spectra were recorded on a Bruker AVANCE NEO 400 spectrometer with an Ascend magnet, an automated tuning 5 mm broadband iProbe, and a 60 position SampleXpress sample changer, or a Bruker AVANCE III 500 spectrometer with an Oxford magnet, an automated tuning 5 mm $^{1}H/^{13}C/^{15}N$ cold probe, and a 24 position SampleCase sample changer interfaced to a UNIX computer using the VnmrJ software. All NMR experiments were performed at ambient temperature. Spectra were processed using MestReNova *v.* 14.2.3 (Mestrelab Research, S.L, Santiago De Compostela, Spain). Chemical shifts for ^{1}H NMR and ^{13}C NMR signals were referenced to the solvent resonance frequencies.

Fourier transform infrared (FTIR) spectra were recorded on an IR Prestige 21 system, equipped with a diamond crystal attenuated total reflection (ATR) accessory (Shimadzu Corp., Japan), and analyzed using IRsolution *v.* 1.40 software. A small amount of sample was placed directly on the ATR crystal for IR measurement.

Ultraviolet-visible (UV-vis) absorption spectra were recorded on a UV-2500 PC spectrophotometer (Shimadzu Corp., Japan) using a 1 cm path length quartz cell at ambient temperature and analyzed using UV Probe *v.* 2.71 software.

Elemental Characterization:

Elemental analyses (C, H, N, and Cl) were conducted by Midwest Microlab (Indianapolis, IN, USA).

Mass Spectrometric Characterization:

Electrospray ionization mass spectrometry (ESI-MS) experiments were performed using a Thermo Scientific Q Exactive Focus. Samples were loop injected (10 μL) and methanol was used as a mobile phase at a flow rate of 600 μL/min. The Q Exactive Focus HESI source was operated in full MS in positive and negative mode. The mass resolution was tuned to 70000 FWHM at m/z 200. The spray voltage was set to 3.5 kV for positive mode and 2.8 kV for negative mode. The sheath gas and auxiliary gas flow rates were maintained at 40 and 10 arbitrary units, respectively. The transfer capillary temperature was held at 320 °C and the S-Lens RF level was set at 50 V. Exactive Series 2.11 /Xcalibur 4.2.47 software was used for data acquisition and processing.

Matrix-assisted laser desorption ionization–time of flight mass spectrometry (MALDI-TOF MS) was performed on a microflex LRF mass spectrometer (Bruker Corporation, Billerica, MA) equipped with a pulsed nitrogen laser (337 nm, 25 kV), and 200 laser shots were collected per spectrum. P(L-Glu)$_{40}$ **2** was analyzed in negative linear mode using α-cyano-4-hydroxycinnamic acid (CHCA) as the matrix (10 mg/mL in a 70:30 acetonitrile:water solution containing 0.1% TFA). The polymer was dissolved in DI water (~1 mg/mL), and the sample and matrix solutions were combined at a 1:20 volumetric ratio. P(L-DOPA(OAc)$_2$)$_{50}$ **6**, in contrast, was analyzed in positive linear mode using trans-2-[3-(4-tert-butylphenyl)-2-methyl-2-propylidene]malononitrile (DCTB) as the matrix and potassium trifluoroacetate (KTFA) as the cationization reagent. The polymer sample (1 mg/mL in THF), DCTB (10 mg/mL in chloroform), and KTFA (10 mg/mL in acetone) were mixed at a 1:20:1 (sample:matrix:cationization reagent) volumetric ratio. For both polymers, 1 μL of the prepared mixture was deposited onto a stainless-steel target plate and air-dried at room temperature prior to measurement.

Size Exclusion Chromatographic Characterization:

DMF-based size exclusion chromatography (SEC) was conducted on a Tosoh HLC-8420 EcoSEC Elite® GPC system with a three-column set of Super AW-L guard column (35 × 4.6

mm), 4 μm TSKgel SuperAW3000 (150 × 6 mm), and 6 μm TSKgel SuperAW4000 (150 × 6 mm).

The system was equilibrated at 50 °C in pre-filtered DMF (containing 0.01 mol/L LiBr), which served as the polymer solvent and eluent, with flow rates of 0.35 mL/min for the sample side and 0.175 mL/min for the reference side. Polymer solutions were prepared at known concentrations (1.0 - 2.0 mg/mL) and filtered through 0.22 μm PTFE syringe filters prior to injection (10 μL). Data collection and analysis were processed with EcoSEC Data Analysis *v.* 2.30 (Tosoh Bioscience). The system was calibrated with PMMA standards (Agilent Technologies, USA) ranging from 2680 to 1568000 Da.

Thermal Characterization:

Thermogravimetric analysis (TGA) was conducted using a Mettler-Toledo TGA2/1100/464 (Mettler-Toledo, Inc., Columbus, OH). The measurements were performed with sample loadings of *ca.* 3 - 5 mg in 100 μL aluminum pans with a heating range of 25 °C - 500 °C and a heating rate of 10 °C/min under N_2 atmosphere. Data were analyzed using Mettler-Toledo STAR^e *v.* 17.00 Software.

Glass transition temperatures (T_g) were measured by differential scanning calorimetry (DSC) on a Mettler-Toledo DSC3/700/1190 under N_2 atmosphere. Measurements were performed on sample masses of *ca.* 3 - 5 mg in 40 μL aluminum pans, with heating and cooling rates of 10 °C/min, and three heating and cooling cycles were conducted. Measurements were analyzed using Mettler-Toledo STAR^e *v.* 17.00 software. The T_g values were determined as the midpoint of the inflection during the second heating scan.

Flammability testing was conducted using a microscale combustion calorimeter (MCC, Fire Testing Technology, East Grinstead, UK) in accordance with ASTM D7309.[1] Solid specimens (*ca.* 3 - 4 mg) were heated from 100 °C to 600 °C at a constant rate of 1 °C/s in a synthetic air atmosphere (20% O_2 / 80% N_2), generated by mixing oxygen (20 mL/min) and nitrogen (80 mL/min). The heat release rate was continuously recorded, from which the peak heat release rate (pHRR) and total heat release (THR), and char yield were determined. The measurements were performed in triplicate.

Electrochemical Characterization:

Electrochemical measurements were conducted using an Autolab PGSTAT204 potentiostat (Metrohm) and analyzed with the Nova 2.1 software. The electrolyte solution (1 M acetate buffer, pH = 5) was sparged with nitrogen for at least 15 min prior to tests, and cyclic voltammetry (CV) data were collected under a blanket of nitrogen at room temperature.

Wide-angle X-ray Scattering (WAXS):

WAXS was performed on a Bruker D8 Bragg–Brentano X-ray powder diffractometer. The sample was placed in the sample holder of a two-circle goniometer, enclosed in a radiation safety enclosure. The X-ray source was a 1 kW Cu X-ray tube, maintained at an operating current of 40 kV and 25 mA. The X-ray optics was the standard Bragg-Brentano para-focusing mode with the X-ray diverging from a DS slit (1mm) at the tube to strike the sample and then converging at a position sensitive X-ray Detector (Lynx-Eye, Bruker-AXS). The two-circle 218 mm diameter q-q goniometer was computer controlled with independent stepper motors and optical encoders for the q circle with the smallest angular step size of 0.0001° 2q. The software suite for data collection and evaluation is windows based. Data collection is automated COMMANDER program by employing a DQL file and analyzed by the program EVA.

400 MHz – DMSO-d_6

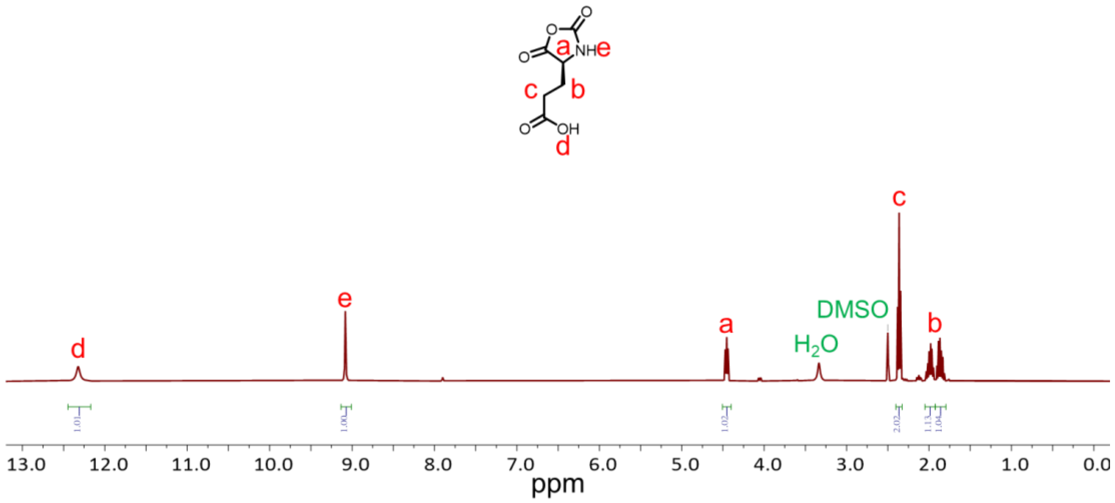

101 MHz – DMSO-d_6

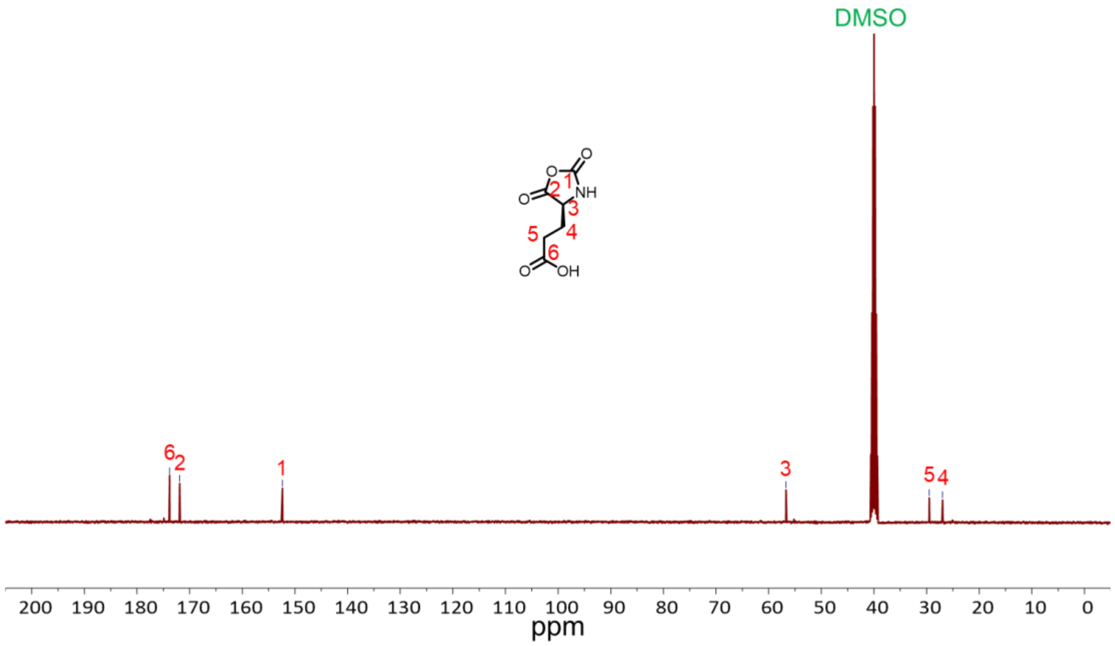

Figure S1. ^{1}H NMR (400 MHz, DMSO-d_6) and ^{13}C NMR (101 MHz, DMSO-d_6) spectra of L-Glu NCA **1**.

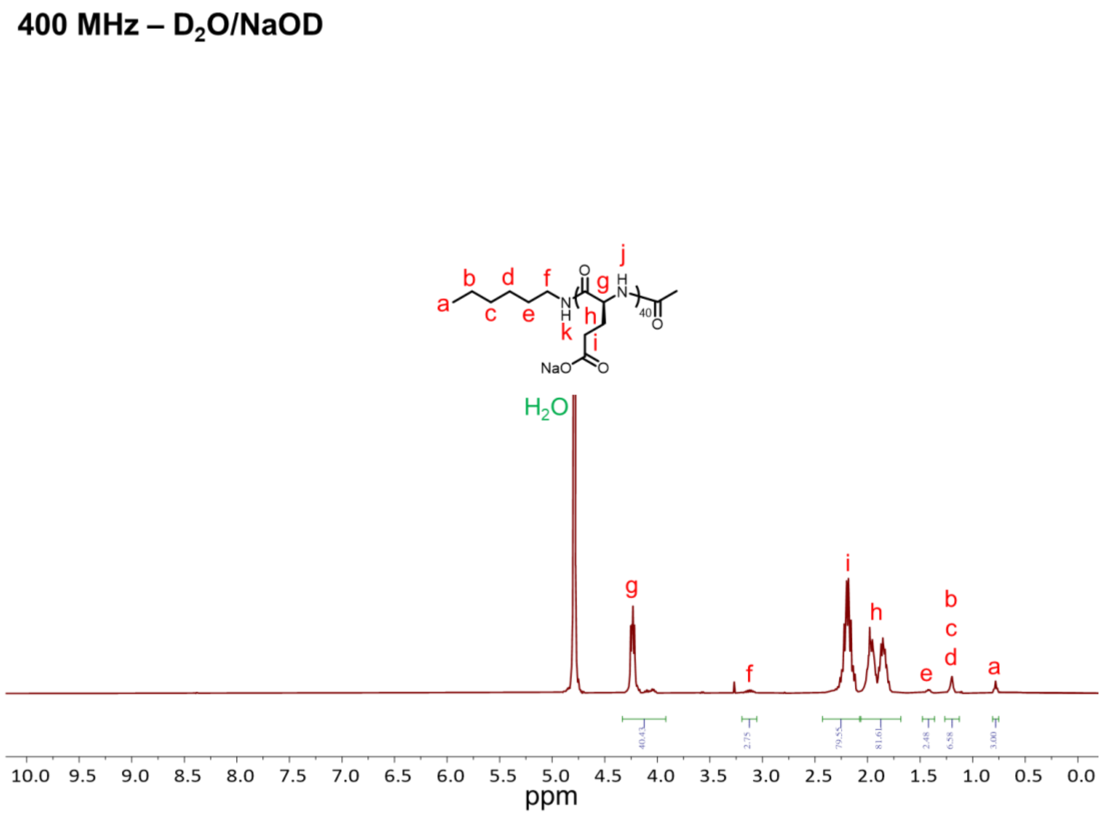

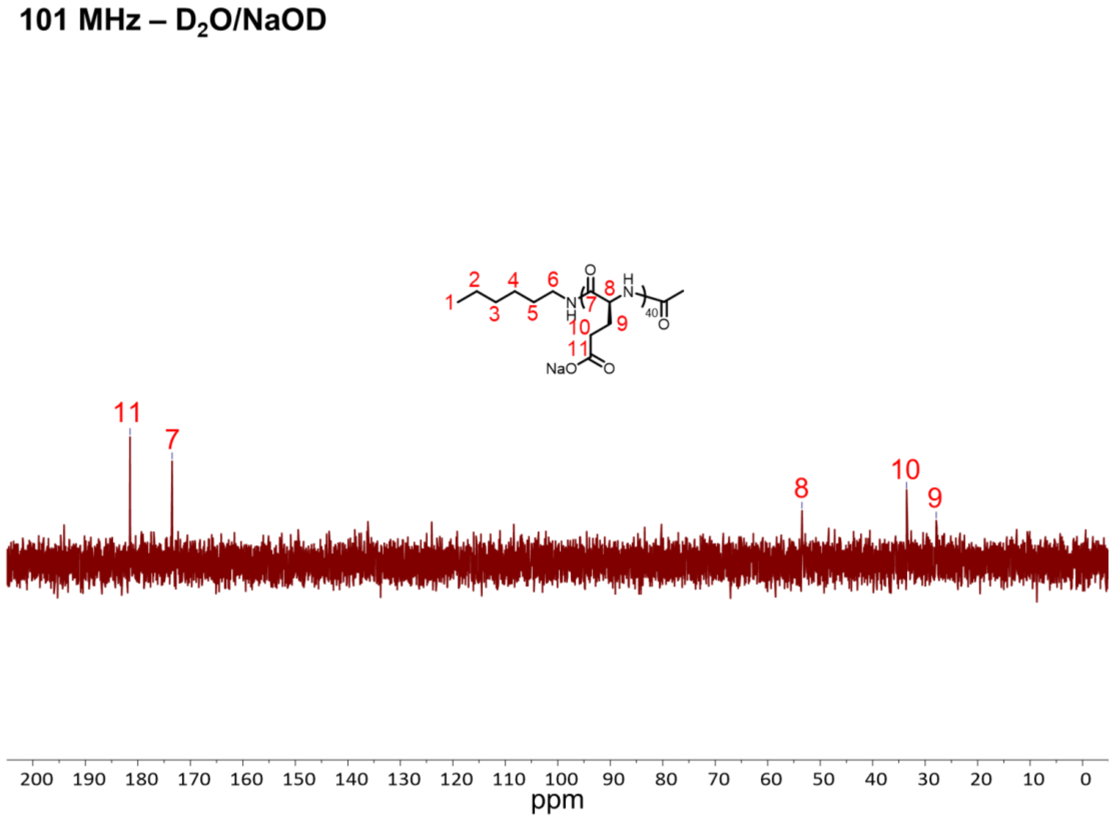

Figure S2. ^{1}H NMR (400 MHz, D_2O/NaOD) and ^{13}C NMR (101 MHz, D_2O/NaOD) spectra of P(L-Glu)$_{40}$ **2**.

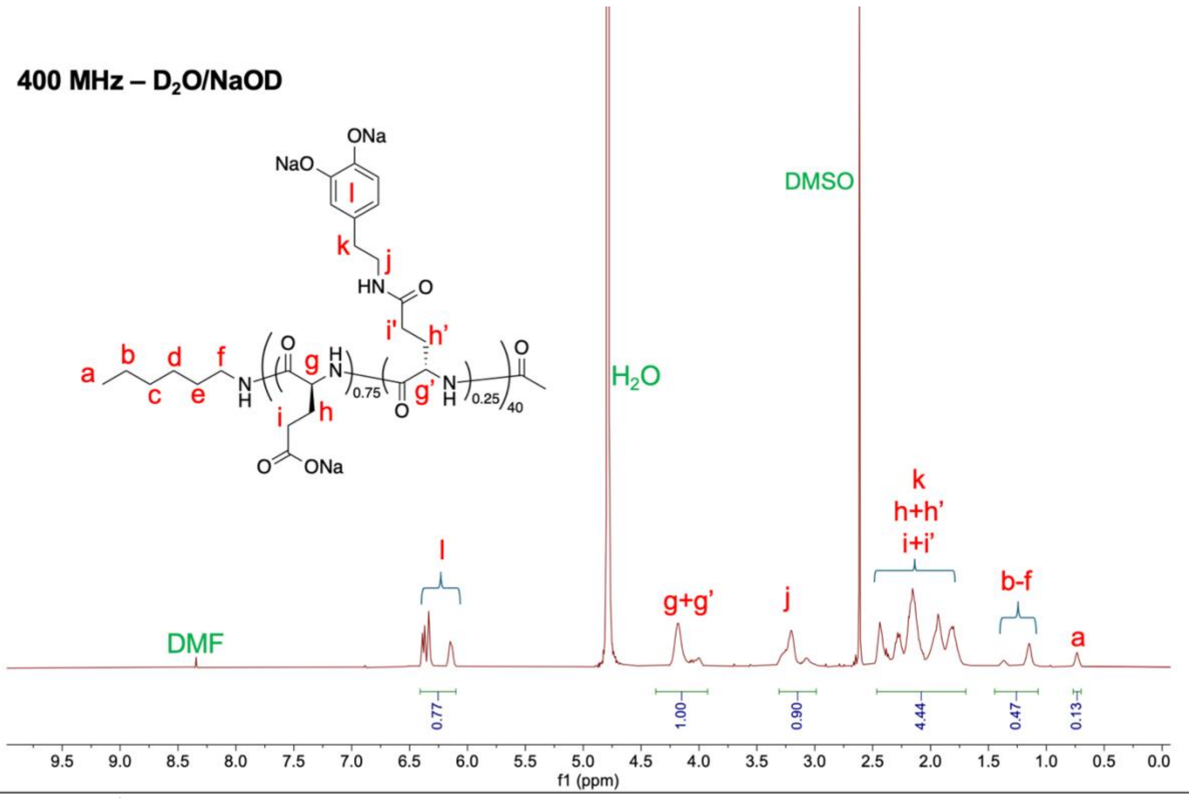

Figure S3. ^{1}H NMR (400 MHz, D_2O/NaOD) spectrum of P(L-Glu)$_{40}$-*g*-DA **3**.

400 MHz – DMSO-d_6

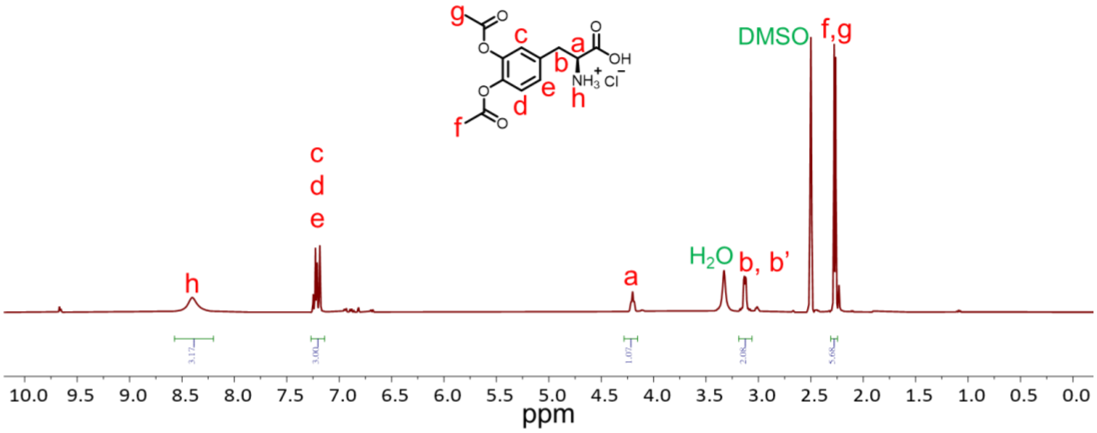

101 MHz – DMSO-d_6

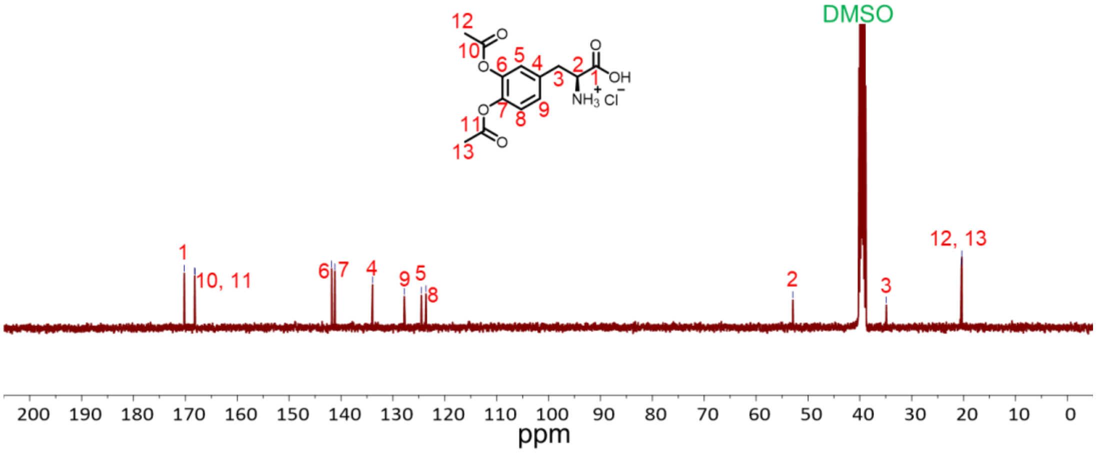

Figure S4. ^1H NMR (400 MHz, DMSO-d_6) and ^{13}C NMR (101 MHz, DMSO-d_6) spectra of L-DOPA(OAc)$_2$ **4**.

400 MHz – DMSO-d_6

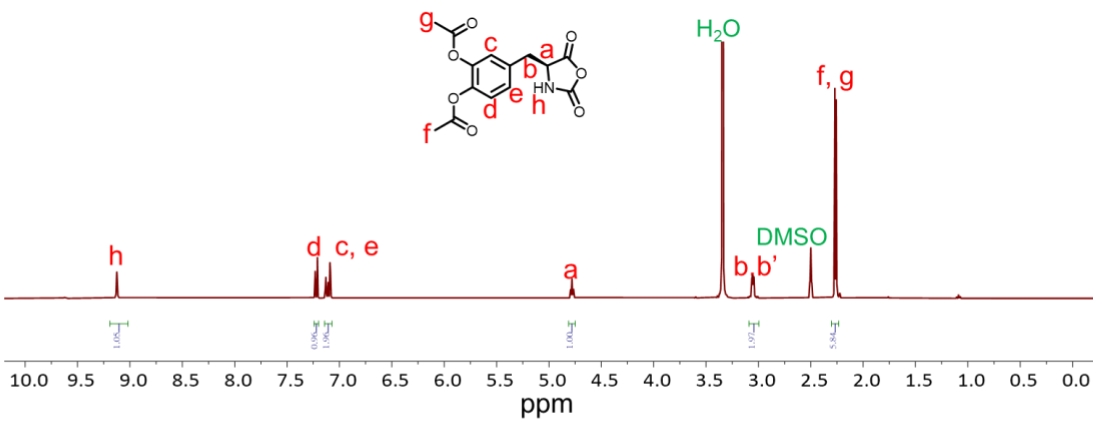

101 MHz – DMSO-d_6

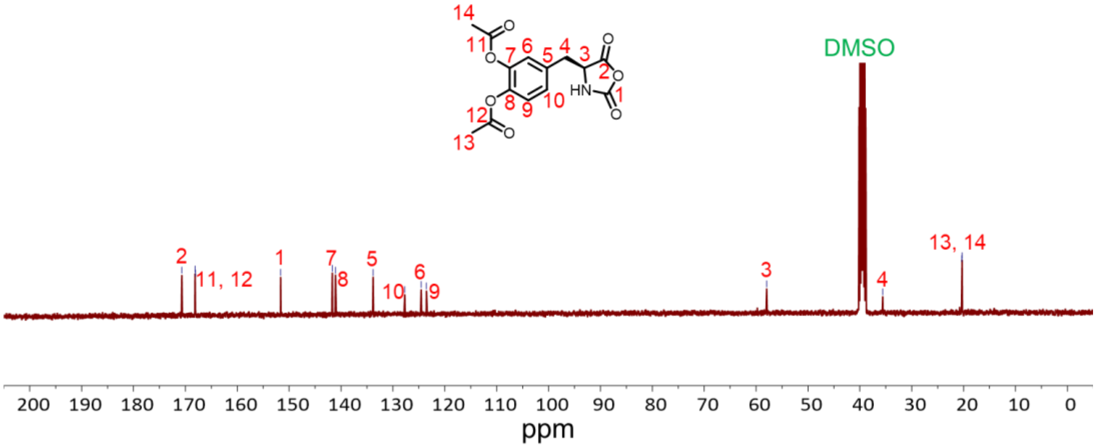

Figure S5. ^{1}H NMR (400 MHz, DMSO-d_6) and ^{13}C NMR (101 MHz, DMSO-d_6) spectra of L-DOPA(OAc)$_2$ NCA **5**.

500 MHz – DMSO-d_6

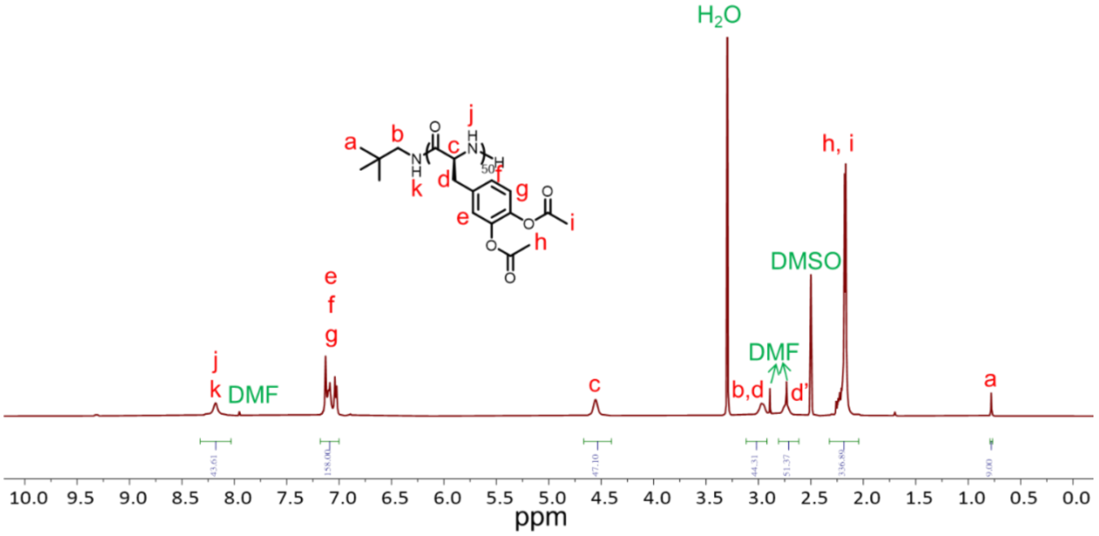

126 MHz – DMSO-d_6

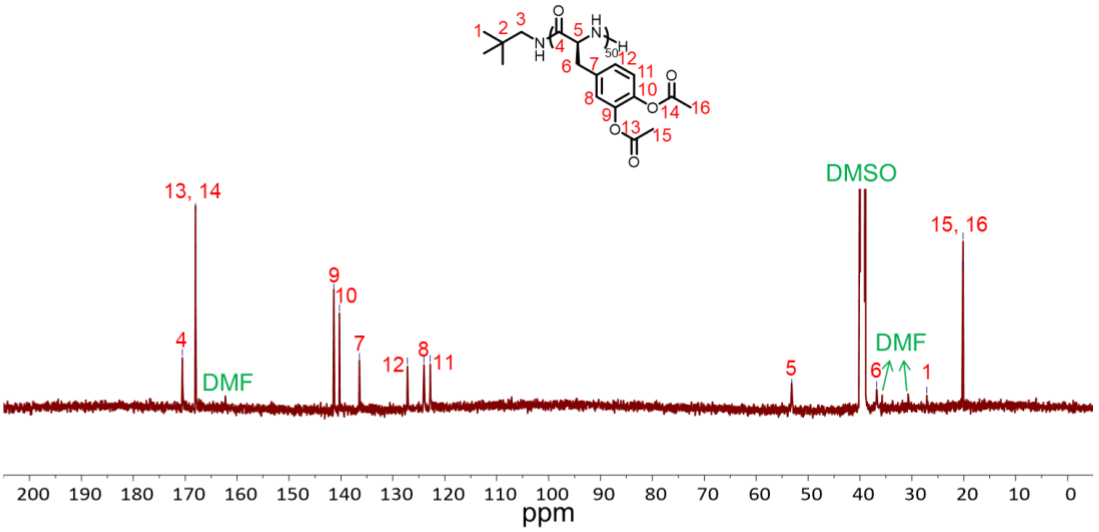

Figure S6. ^{1}H NMR (500 MHz, DMSO-d_6) and ^{13}C NMR (126 MHz, DMSO-d_6) spectra of P(L-DOPA(OAc)$_2$)$_{50}$ **6**.

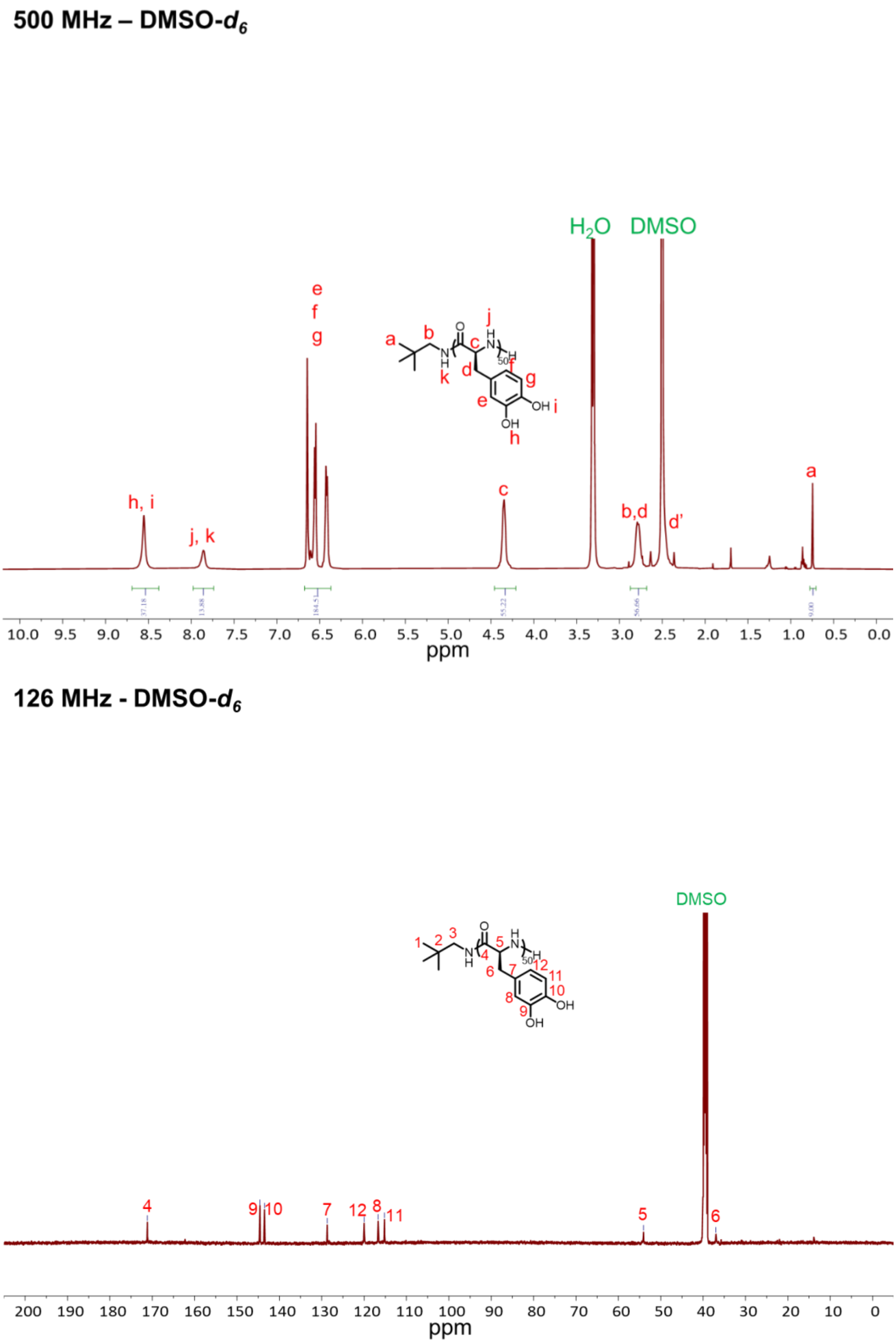

Figure S7. ^{1}H NMR (500 MHz, DMSO-d_6) and ^{13}C NMR (126 MHz, DMSO-d_6) spectra of P(L-DOPA)$_{50}$ **7**.

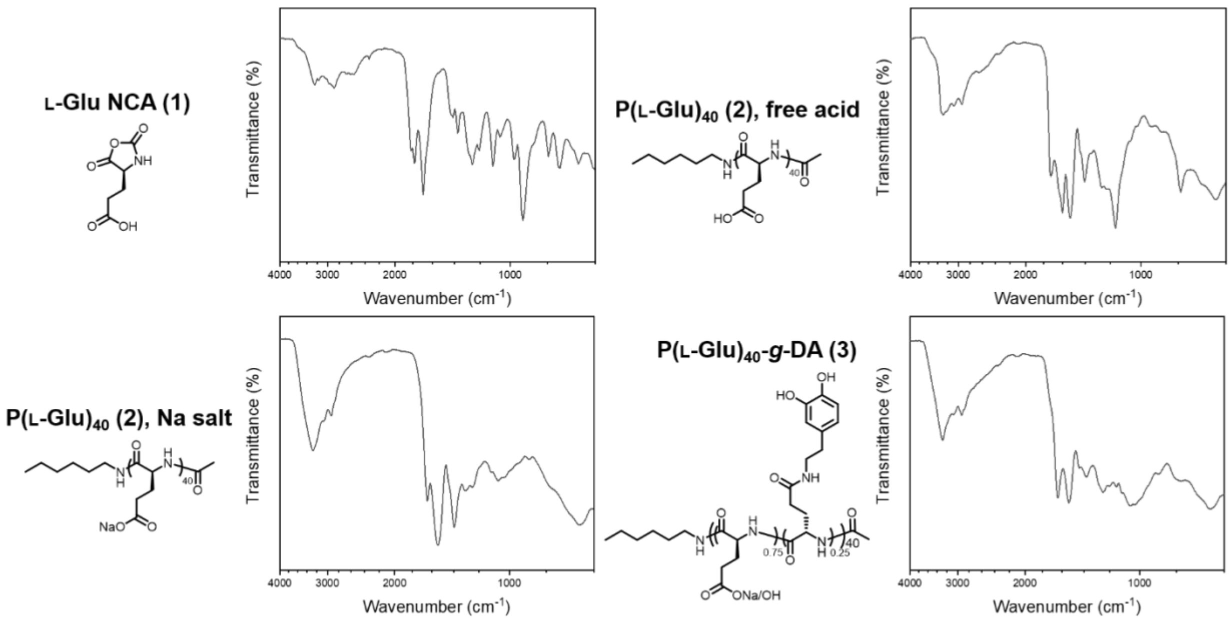

Figure S8. ATR-FTIR spectra of L-Glu NCA (**1**), P(L-Glu)$_{40}$ **2** and P(L-Glu)$_{40}$-*g*-DA **3**.

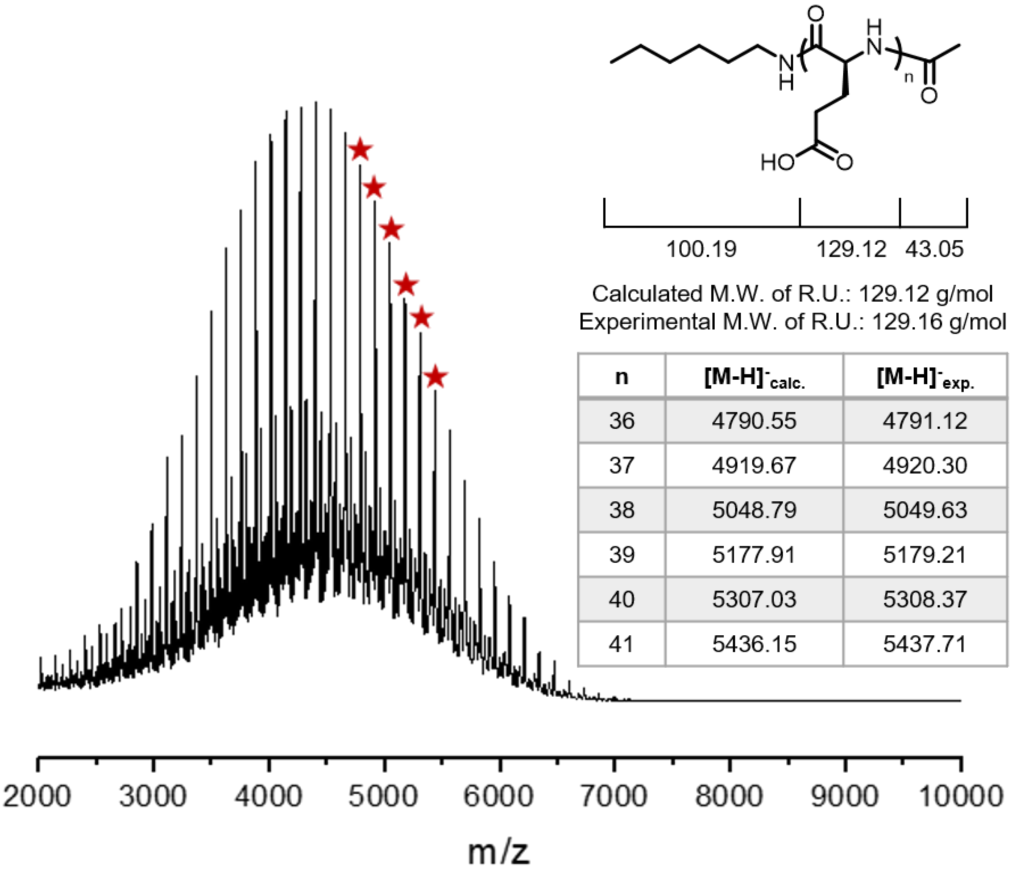

n	$[M-H]^-_{calc.}$	$[M-H]^-_{exp.}$
36	4790.55	4791.12
37	4919.67	4920.30
38	5048.79	5049.63
39	5177.91	5179.21
40	5307.03	5308.37
41	5436.15	5437.71

Figure S9. MALDI-ToF spectrum of P(L-Glu)$_{40}$ **2**, with the data contained within the table noted by red stars upon the spectrum.

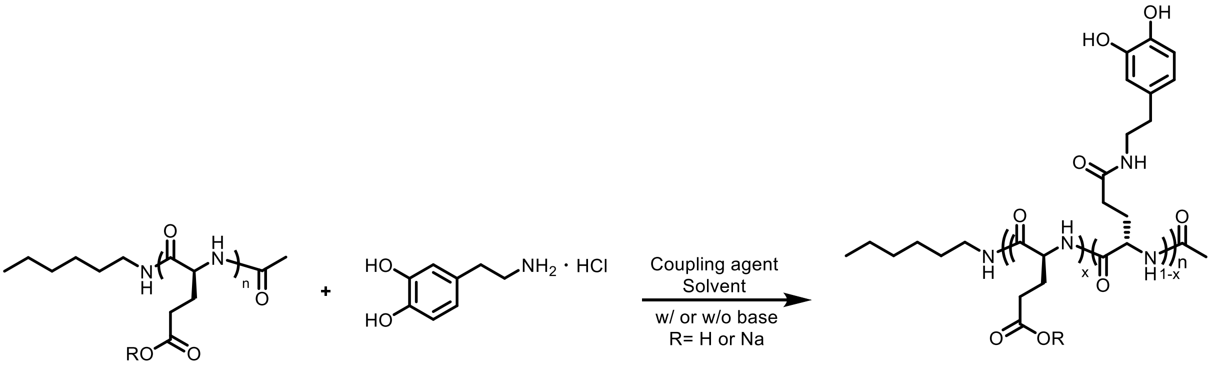

Figure S10. General synthetic scheme for constructing a catechol-functionalized polypeptide by side chain amidation of P(L-Glu)$_{40}$ **3** with dopamine.

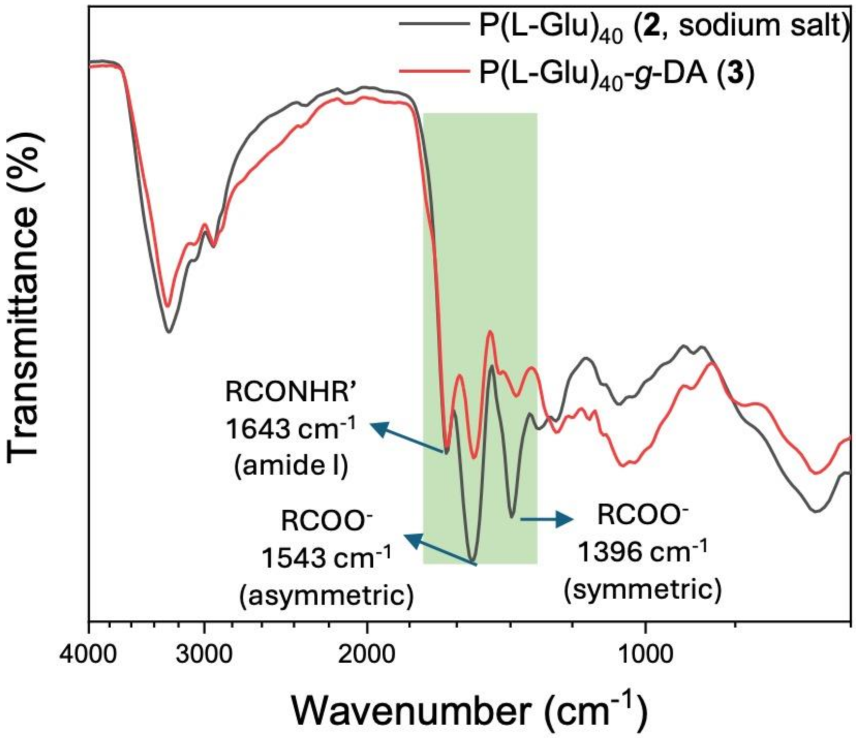

Figure S11. ATR-FTIR spectra of P(L-Glu)$_{40}$ (**2**, Na salt) and P(L-Glu)$_{40}$-*g*-DA **3**. The highlighted region shows the decreased intensities of carboxylate peaks following amidation, indicating successful grafting of dopamine.

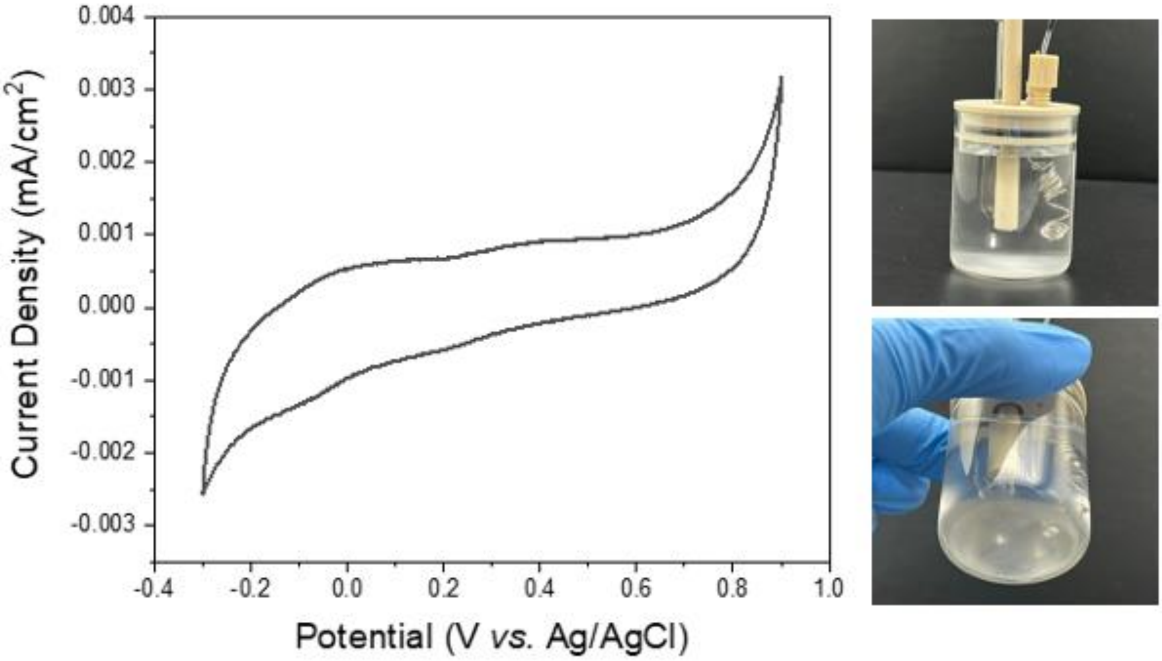

Figure S12. Cyclic voltammogram for P(L-Glu)$_{40}$-*g*-DA **3** in aqueous solution (0.5 mg/mL suspended in 1 M acetate buffer, pH = 5) collected at a scan rate of 10 mV·s^{-1}.

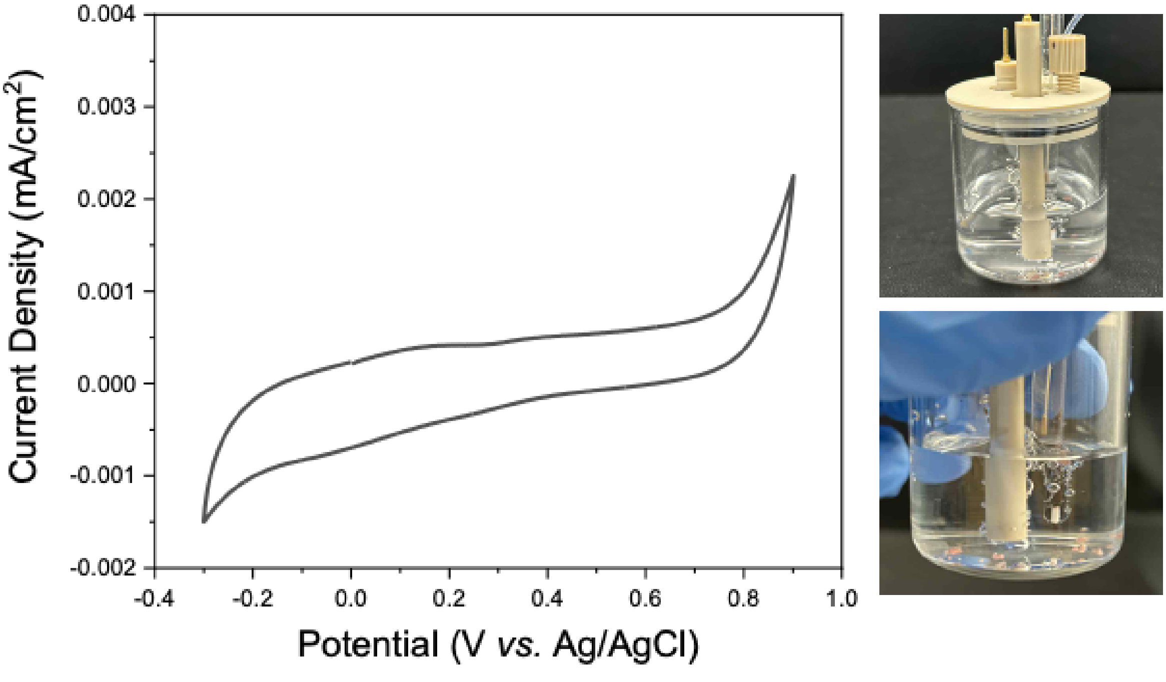

Figure S13. Cyclic voltammogram for P(L-DOPA)$_{50}$ **7** in aqueous solution (0.5 mg/mL suspended in 1 M acetate buffer, pH = 5) recorded at a scan rate of 10 mV·s^{-1}.

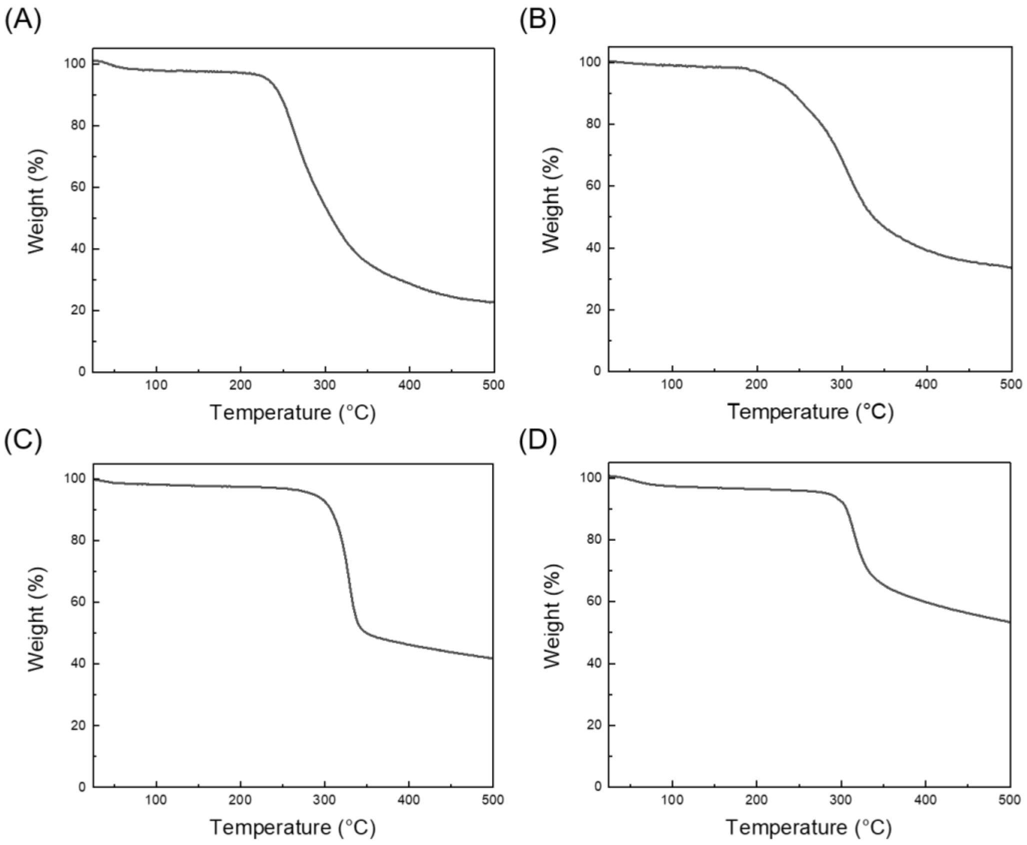

Figure S14. TGA traces of polypeptides (A) P(L-Glu)$_{40}$ **2** (free acid), (B) P(L-Glu)$_{40}$-*g*-DA **3**, (C) P(L-DOPA(OAc)$_2$)$_{50}$ **6**, and (D) P(L-DOPA)$_{50}$ **7**.

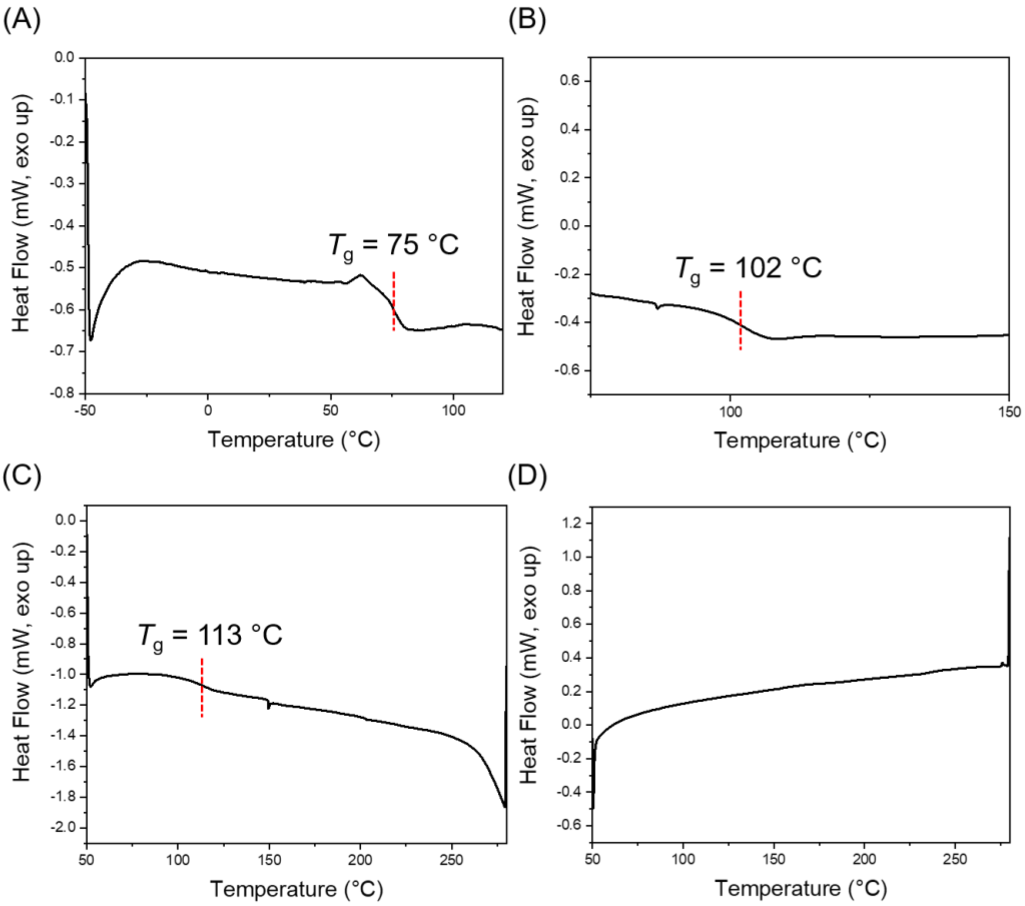

Figure S15. DSC traces of polypeptides (A) P(L-Glu)$_{40}$ **2** (free acid), (B) P(L-Glu)$_{40}$-*g*-DA **3**, (C) P(L-DOPA(OAc)$_2$)$_{50}$ **6**, and (D) P(L-DOPA)$_{50}$ **7**. The DSC data were reported from the second heating cycles.

	M_n[a] (kDa)	DP_n[a]	M_n[b] (kDa)	M_w[b] (kDa)	$Đ$[b]
**P(L-DOPA(OAc)$_2$)$_{50}$**	13.2	50	7.52	11.2	1.50

[a]determined by ^{1}H NMR [b]determined by DMF SEC

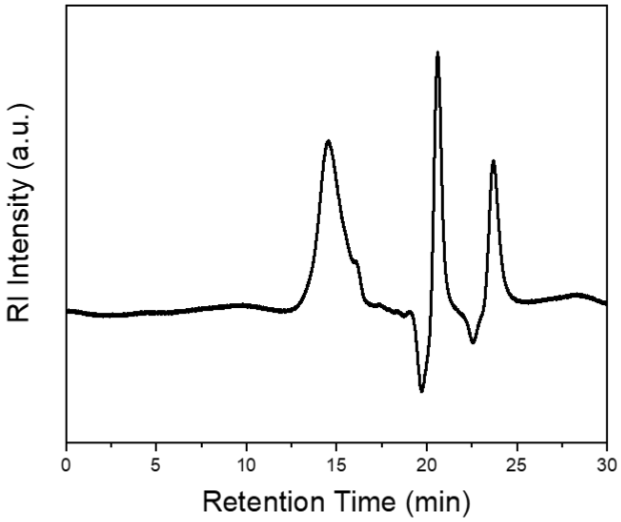

Figure S16. Table including the values of number-average molar mass (M_n) and number-average degree of polymerization (DP_n) as determined by ^{1}H NMR spectroscopy, and number-average molar mass (M_n), weight-average molar mass (M_w), and dispersity ($Đ$) of P(L-DOPA(OAc)$_2$)$_{50}$ **6**, as determined by SEC eluting with DMF containing 0.01 M LiBr, and calibrated using poly(methyl methacrylate) standards. SEC trace for P(L-DOPA(OAc)$_2$)$_{50}$ eluting with DMF containing 0.01 M LiBr at a flow rate of 0.35 mL/min for the sample and 0.175 mL/min for the reference.

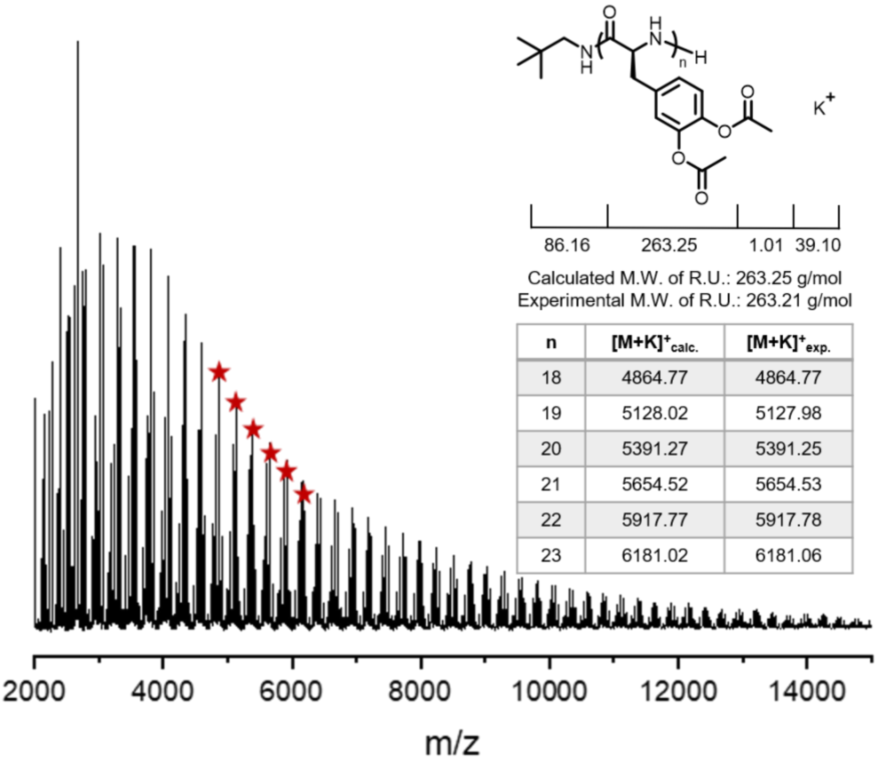

n	$[M+K]^+_{calc.}$	$[M+K]^+_{exp.}$
18	4864.77	4864.77
19	5128.02	5127.98
20	5391.27	5391.25
21	5654.52	5654.53
22	5917.77	5917.78
23	6181.02	6181.06

Figure S17. MALDI-ToF spectrum of polypeptide P(L-DOPA(OAc)$_2$)$_{50}$ **6**, with the data contained within the table noted by red stars upon the spectrum.

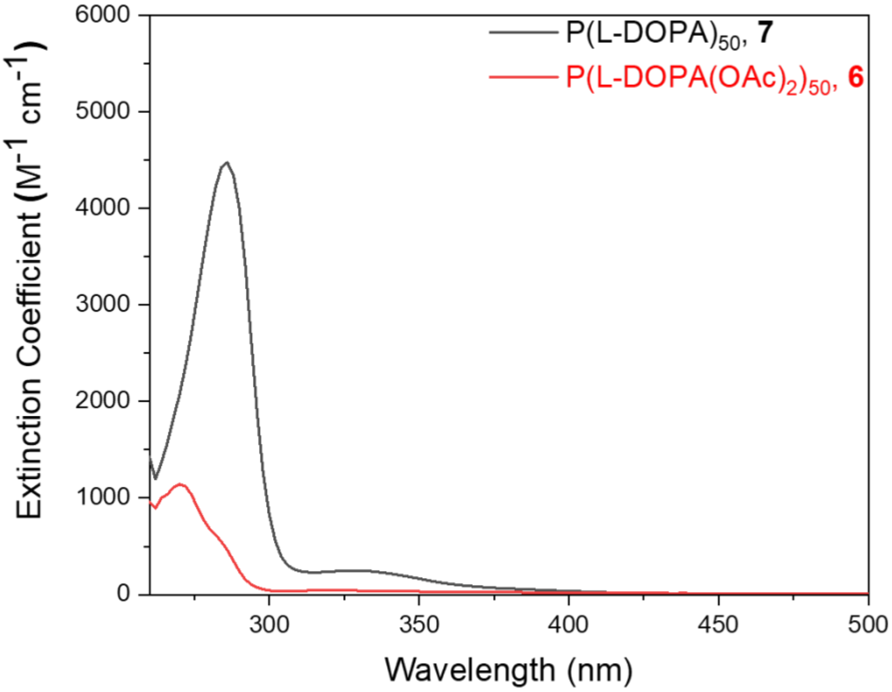

Figure S18. The UV-vis absorption spectra of P(L-DOPA(OAc)$_2$)$_{50}$ **6** and P(L-DOPA)$_{50}$ **7** in DMF, plotted as extinction coefficient *vs*. wavelength to illustrate significantly higher intensities for both the $\pi \rightarrow \pi^*$ and $n \rightarrow \pi^*$ transitions for **7** *vs* **6**.

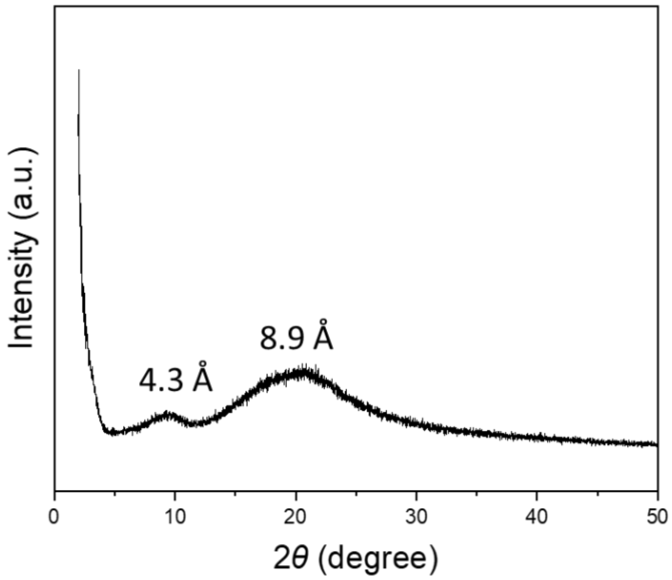

Figure S19. WAXS pattern of P(L-DOPA)$_{50}$ **7**.

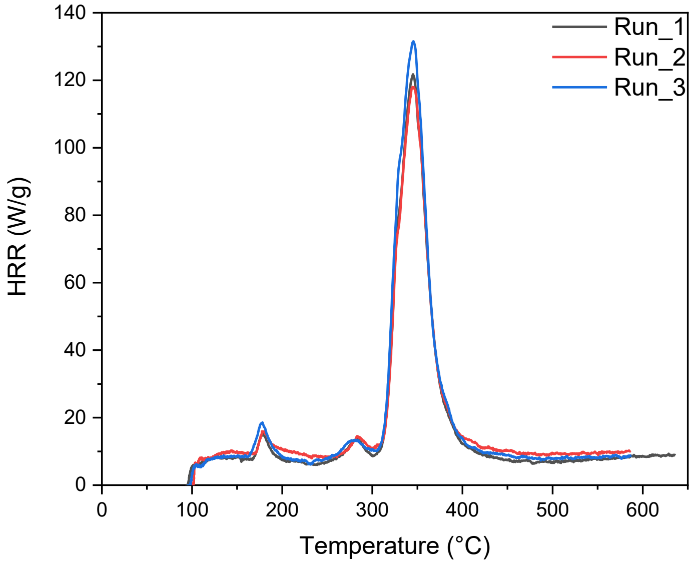

Figure S20. Microscale combustion calorimetry (MCC) curves of P(L-DOPA)$_{50}$ **7** in triplicate, measuring heat release rate (HRR) as a function of temperature with a heating of 1 °C/s in a synthetic air atmosphere (20% O_2 / 80% N_2).

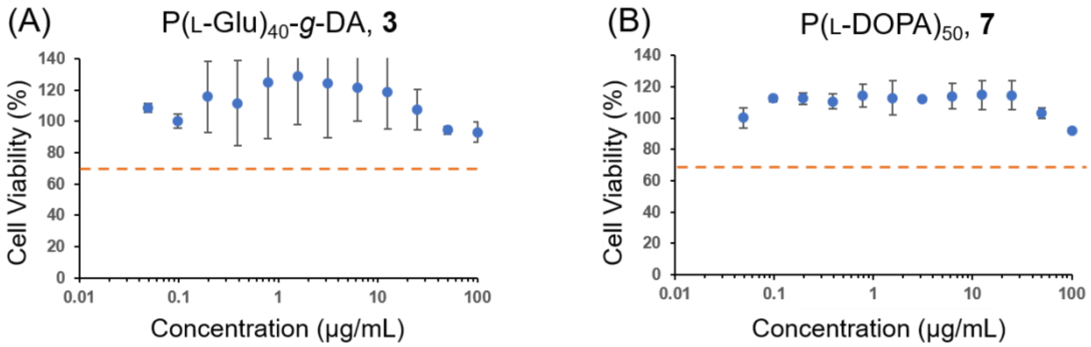

Figure S21. Cytotoxicity effects of P(L-Glu)$_{40}$-*g*-DA **3** and P(L-DOPA)$_{50}$ **7** on fibroblast (NIH/3T3) cells. The orange dotted line in the plots indicates 70% cell viability level.

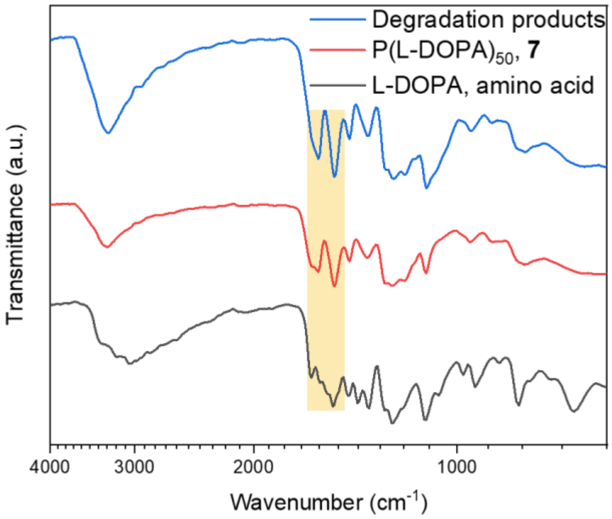

Figure S22. ATR-FTIR spectra of polypeptide **7** before and after acidic degradation. Comparison of the FTIR spectra of pristine polypeptide **7** (red), its corresponding degradation mixture after treatment in 6 M HCl (blue), and a reference spectrum of L-DOPA (black). The similarities of the amide absorption band (highlighted region) in both the pristine polypeptide and the degraded sample indicate incomplete hydrolysis of the polypeptide backbone, and characteristic features of free L-DOPA (expected degradation product) are not predominant.

Table S1. Data table of the amidation reaction conditions and degrees of incorporation for conjugation of dopamine into P(L-Glu)$_{40}$ **3**.

Entry	R	Coupling Agent[a]	Dopamine HCl (eq.)	Solvent	Base	Conditions[b]	Degree of Incorporation (%)[c]
1	H	EDC[2] (2 eq.)	2	Dry DMF	TEA (1 eq.)	1. 0 °C, 20 min<br>2. 0 °C to r.t., 10 h	-
2	H	DMTMM[3] (1.5 eq.)	3	Acetate buffer (0.1 M, pH = 5)	-	1. r.t., 15 min<br>2. Dopamine HCl, r.t., 24 h	-
3	Na	EDC/NHS[4] (1.2 eq./2.4 eq.)	1.2	DI water	-	1. 0 °C, 30 min; 0 °C to r.t., 1.5 h<br>2. Dopamine HCl, r.t., 19 h	25
4	Na	EDC/HOBt (1.2 eq./2.4 eq.)	1.2	DI water	TEA (1.2 eq.)	1. 0 °C, 30 min; 0 °C to r.t., 1.5 h<br>2. Dopamine HCl, r.t., 19 h	14
5	Na	EDC/NHS (1 eq./2 eq.)	1	Acetate buffer (0.1 M, pH = 5)	-	1. 0 °C, 30 min; 0 °C to r.t., 1.5 h<br>2. Dopamine HCl, r.t., 22 h	19

[a]Based on literature reports,[2-4] various coupling agents were attempted during amidation reactions. EDC = 1-ethyl-3-(3'-dimethylaminopropyl)carbodiimide hydrochloride; DMTMM = 4-(4,6-dimethoxy-1,3,5-triazin-2-yl)-4-methylmorpholinium chloride; NHS = *N*-hydroxysuccinimide; HOBt = hydroxybenzotriazole.

[b]For entry 1, P(L-Glu)$_{40}$ **3**, coupling agent, dopamine HCl, solvent, and base (TEA) were added in one portion, and the reaction mixture was stirred for 20 min at 0 °C. After warming to r.t., it was allowed to react for another 10 h. For entries 2 to 5, P(L-Glu)$_{40}$ **3** was first equilibrated with the coupling agent in an ice bath for 30 min, then allowed to warm up to room temperature with stirring for another 1.5 h to sufficiently activate the side-chain carboxylic acids (entry 2) or carboxylates (entries 3-5). Dopamine HCl was subsequently added, with base included only for entry 4, and the reaction mixture was stirred at room temperature overnight (19 h for entries 3 and 4; 22 h for entry 5). All reactions were purified by dialysis against nanopure water. For entry 3, additional precipitation steps were performed, as described in the synthesis section, to remove residual small-molecule impurities.

[c]The degree of dopamine incorporation was determined from ^{1}H NMR spectroscopy, by comparing the integration ratio of the aromatic protons of the catechol side chains to the methine protons of P(L-Glu)$_{40}$ **3**. Due to the low conjugation in entries 1 and 2 (<5%), quantification by ^{1}H NMR spectroscopy was infeasible.

Table S2. Data table details for microscale combustion calorimetry (MCC) results for the L-DOPA polypeptide **7**. Reported values include peak heat release rate (PHRR), total heat release (THR), char yield, and the temperature at peak heat release rate (T_{max}). Measurements were conducted in triplicate, and values shown each individual test.

Test #	PHRR (W/g)	THR (kJ/g)	Char Yield (%)	T_{max} (°C)
1	113.4	4.6	45.9	345.1
2	107.4	4.3	43.3	344.5
3	121.4	4.8	50.0	345.5

References

1. ASTM D7309-21: Standard Test Method for Determining Flammability Characteristics of Plastics and Other Solid Materials Using Microscale Combustion Calorimetry. ASTM International: West Conshohocken, PA, 2021.

2. Dong, M.; Song, Y.; Wang, H.; Su, L.; Shen, Y.; Tran, D. K.; Letteri, R. A.; Flores, J. A.; Lin, Y.-N.; Li, J.; Wooley, K. L., Degradable sugar-based magnetic hybrid nanoparticles for recovery of crude oil from aqueous environments. *Polym. Chem.* **2020,** *11* (30), 4895-4903.

3. Ochs, C. J.; Hong, T.; Such, G. K.; Cui, J.; Postma, A.; Caruso, F., Dopamine-Mediated Continuous Assembly of Biodegradable Capsules. *Chem. Mater.* **2011,** *23* (13), 3141-3143.

4. Zhang, R.-L.; Xu, S.; Luo, J.; Shi, D.-J.; Liu, C.; Liu, X.-Y., One-pot green synthesis of nanohybrid structures: gold nanoparticles in poly(γ-glutamic acid) copolymer nanoparticles. *RSC Adv.* **2014,** *4* (48), 25106-25113.
